# The interaction between microbiota and immune in intestinal inflammatory diseases: Global research status and trends

**DOI:** 10.3389/fcimb.2023.1128249

**Published:** 2023-02-07

**Authors:** Chuan Liu, Wenhao Su, Zongbiao Tan, Jixiang Zhang, Weiguo Dong

**Affiliations:** Department of Gastroenterology, Renmin Hospital of Wuhan University, Wuhan, Hubei, China

**Keywords:** microbiota, immune, intestinal inflammatory diseases, bibliometric, CiteSpace

## Abstract

**Aims:**

This study aimed to conduct a bibliometric analysis of the relevant literature on the interaction between microbiota and immune in intestinal inflammatory diseases, and show its current status, hotspots, and development trends.

**Methods:**

The related literature was acquired from the Web of Science Core Collection on October 12, 2022. Co-occurrence and cooperation relationship analysis of authors, institutions, countries, references, and keywords in the literature were carried out through CiteSpace 6.1.R3 software and the Online Analysis platform of Literature Metrology. At the same time, the relevant knowledge maps were drawn, and the keywords cluster analysis and emergence analysis were performed.

**Results:**

3,608 related publications were included, showing that the number of articles in this field is increasing year by year. The results showed that Gasbarrini A and Sokol H were the authors with the highest cumulative number of articles with 25, and the institution with the most articles was Harvard University with 142 articles. The USA was far ahead in the article output, with 1,131 articles, and had a dominant role, followed by China with 707 articles. The journal Frontiers in Immunology contributed the most to this research field with 213 articles. In the cooperation network analysis, the USA, Harvard University, and Xavier RJ were the most widely collaborated country, institution, and author, respectively, which implied a high level of influence. Keywords analysis showed that there were 770 keywords, which were mainly classified as internal related diseases, such as “inflammatory bowel disease”, “irritable bowel syndrome”, “colorectal cancer”, and the mechanism of interaction of microbiota and immune, such as “intestinal microbiota”, “commensal microbiota”, “regulatory T cell”, “dendritic cell”, “barrier function”, “activation”, “anti-inflammatory properties”, “intestinal epithelium”, and “diversity”. Emerging analysis showed that future research hotspots and trends might be the short-chain fatty acid, gut dysbiosis, gut-liver axis, and fusobacterium nucleatum.

**Conclusion:**

This research was the first bibliometric analysis of publications in the field of interaction between microbiota and immune in intestinal inflammatory diseases using visualization software and data information mining, and obtained the current status, hotspots, and development of this field, which provides a theoretical basis for its scientific research.

## 1 Introduction

Intestinal inflammatory diseases are the common diseases of intestinal tissues that seriously disturb human life and health, mainly manifesting as clinical symptoms such as digestive dysfunction, abdominal pain, diarrhea, and hematochezia caused by damaged intestinal tissues. The incidence of intestinal inflammatory diseases, such as inflammatory bowel disease (IBD), celiac disease, necrotizing small bowel colitis (NEC), and systemic autoimmune diseases, is increasing worldwide (Kaplan and Windsor, 2021; Lebwohl and Rubio-Tapia, 2021; Aziz et al., 2022). Among them, IBD is the most common intestinal inflammatory disease, containing ulcerative colitis (UC) and Crohn’s disease (CD), and its prevalence is expected to reach 1% by 2030, which will cause a significant global burden on healthcare resources (Kaplan and Windsor, 2021). Celiac disease is an immune-mediated chronic small intestinal malabsorption syndrome caused by ingestion of bran substances in susceptible individuals, and its incidence and prevalence have increased over time (Lebwohl and Rubio-Tapia, 2021). NEC is a serious intestinal inflammatory disease commonly seen in preterm infants, associated with prematurity, infection, hypoxia and dysbiosis of the intestinal flora, and driven by an unspecified inflammatory pathway, with high morbidity and mortality (Aziz et al., 2022). The pathogenesis of intestinal inflammatory diseases may be mainly due to an undeveloped or disturbed intestinal barrier. The intestinal barrier includes external physical, chemical and microbial barriers, and internal immune barriers, which play an important role in the intestine and are essential for human health (Mehandru and Colombel, 2021).

Numerous studies have shown that the interaction between the gut microbiota and immune cells plays a central role in intestinal inflammation and immune regulation (Round and Mazmanian, 2009; Hooper et al., 2012; Caruso et al., 2020; Xue et al., 2020; Zheng et al., 2020). The interaction between the natural immune system of the mucosa and the microbiota facilitates the immune regulation of the intestinal ecosystem (Round and Mazmanian, 2009). A typical feature of natural immunity is the recognition of potentially pathogenic bacteria and harmless antigens through receptor recognition patterns (PRRs), Toll-like receptors (TLRs) being the main class of PRRs expressed on macrophages, neutrophils, dendritic cells, intestinal epithelial cells (IECs) and other immune cells. The intestinal epithelial cells are the most important part of the natural defense mechanisms of the mucosal surface, which are directly involved in various immune processes in addition to their absorptive, digestive and secretory functions (Parikh et al., 2019). Another important immune part of the intestine is the lamina propria, where a large number of macrophages, dendritic cells, T cells and IgA-secreting B cells are present. Intrinsic layer B cells are active and can be converted into IgA-producing plasma cells. IgA is secreted into the intestinal lumen after transport through the epithelium. Specific intestinal epithelial cells take up antigens in the intestinal lumen and transport them to specific antigen-presenting cells (APCs) that trigger the acquired immune system(Zheng et al., 2020; Chen et al., 2021). In general, the intestinal microbiota regulates the formation and maturation of the intestinal immune system against pathogenic bacterial invasion. In particular, early in life, the microbiota induces the intestinal immune system to recognize harmless and harmful bacteria, establish a balance between them, and maintain intestinal health. When this balance is altered, multiple gastrointestinal and extraintestinal diseases occur (Mehandru and Colombel, 2021). Therefore, exploring the interaction between the microbiota and the immune system in inflammatory diseases of the intestine is an important topic.

Bibliometrics, which combines mathematics, statistics, and literature, uses quantitative analysis to explore the structural features and hot trends of the discipline and to evaluate and predict results (Wang et al., 2014). Thanks to the use of computer tools, the discipline has gained higher attention in terms of theory and application, and on this basis, scientific knowledge mapping, which composes the literature, is widely used. As an effective tool for literature analysis, Bibliometrics has been extensively used in various fields of medicine and the number of related bibliometrics has increased exponentially over the years (Li et al., 2022; Liu et al., 2022). However, there is no visual quantitative analysis available for the research of the interaction between microbiota and immune in intestinal inflammatory diseases. Therefore, this paper adopts a bibliometric approach to systematically review the research in this field, and analyzes the research hotspots and development frontiers of this research field with the help of visual literature analysis tools while quantitatively analyzing the relevant literature. Compared with traditional literature reviews, this study is a new attempt to review and visualize the development of this research field on a large time scale from a global perspective, in order to help related scholars keep abreast of the current status and development trend of this field worldwide and provide some guidance for scientific researchers and policymakers to carry out related work.

## 2 Materials and methods

### 2.1 Data source

Data were acquired from the Web of Science Core Collection (WoSCC) of Clarivate Analytics (https://clarivate.com/) on October 12, 2022. The search time range was not limited, and the search was limited to publications written in the English language. In the WoSCC database, the subject term search method was used, and the subject area was not limited. The search terms included “microbiota”, “immune” and “intestinal inflammatory diseases”, among others. All electronic searches were performed on October 12, 2022. The literature was read independently by 2 evaluators, and firstly the initial screening was done based on the title and abstract of the article, and then it was screened again based on the inclusion and exclusion criteria. The inclusion criterion was any article or review related to the topic of the interaction between microbiota and immune in intestinal inflammatory diseases. After the screening, if there was any dispute, the final decision was made by a third evaluator who read through the whole article together and discussed it.

### 2.2 Data establishment and processing

#### 2.2.1 Data creation and conversion

The retrieved journal publications studying the interaction between microbiota and immune in intestinal inflammatory diseases were imported into Note Express software and duplicates were electronically removed and further screened for the final inclusion of 3608 papers. Document data exported from WoSCC were saved in “RefWorks” format. Two researchers reviewed the selected articles, and the following data were identified and recorded for analysis: ①titles, ②authors, ③citation number, ④keywords, ⑤publication year, ⑥institutions, ⑦countries, and ⑧references. In addition, the journal name, the impact factor (IF) and journal ranking (Q1-Q4: Quartile in category) were also recorded using the 2021 edition of the Journal Citation Reports (JCR). Data were converted to “txt” format, named download_*.txt, and then imported into CiteSpace 6.1.R3 version and the Online Analysis platform of Literature Metrology (OALM) (http://bibliometric.com/) for analysis. OALM provides researchers with bibliometric analysis of scientific citation data in the form of graphic visualization through web services, and provides valuable reference information for researchers to carry out research with the simplest operation method and the most intuitive expression method.

#### 2.2.2 Data processing

Excel software was used to analyze the included literature data. We selected appropriate parameters in the CiteSpace 6.1.R3 version to generate co-occurrence visualization maps of authors, institutions, and countries, and performed cluster analysis and emergence analysis of keywords. The co-occurrence chart contains many nodes. Different nodes represent various elements, such as author, country and keywords, etc., while the size of nodes reflects the frequency or importance of elements, and the connection between nodes represents cooperation, co-occurrence, or co-citation. The color of the link represented the time when the node appeared, with cool colors appearing earlier and warm colors appearing later. (1) Time slicing: 1990-2022; time zone selection (year per slice): 1 year; node type: author, institution, country, keywords. (2) The threshold (top N per slice): 10%, that is, the top 10% but less than 100 high-frequency keywords were selected in each time zone. To prevent the co-citation network from being too complex, a Pathfinder algorithm was used in this paper, which could simplify the network by removing the edges that violate the triangle inequality and accurately extract the key structure of the network. The default system was selected for visualization. At the same time, OALM was used to analyze the number of common national articles by year, the number of common keywords by year, partnerships (including authors, institutions, and countries) and article citation relationships.

### 2.3 Literature measure indicators

#### 2.3.1 Frequency

Frequency is one of the metrics of bibliometric analysis, which refers to the number of occurrences of different node types in the analyzed data of a certain field, and the current status of research in this field can be measured and analyzed by counting the high or low frequency of a certain node type.

#### 2.3.2 Centrality

Centrality is one of the main metrics used in network analysis, and is a measure of the status of individuals in a network. Centrality is a central measure of the degree of control of resources by network nodes in a network graph, and mainly measures the role of each node in a particular network graph. In a co-occurrence network, if the centrality of a node is higher, it indicates that the more the node appears on the shortest path in the network graph, the greater the possibility of other nodes establishing co-occurrence relationships with it, and the greater the influence and importance of the node in the network graph. A node with centrality over 0.1 is called a critical node.

#### 2.3.3 Degree

The degree is another common measure of node importance and is the most direct measure of centrality in network analysis. The higher the degree of a node, the more important the node is in the network.

#### 2.3.4 Sigma

Σ is a measure of scientific innovativeness and is used to identify innovative scientific literature. Σ is a metric in CiteSpace that combines importance in space (centrality) and importance in time (burst), so the size of Σ is directly related to the size of centrality and burst, and the larger the value of the latter two, the larger the value of Σ. If a node has a higher Σ value, it indicates a higher level of innovation.

#### 2.3.5 Burst

The burst detection algorithm is used in the metrological analysis of network graphs to determine the sudden growth rate of nodes in a certain period, and to judge the surge period and the degree of the surge of a certain node based on the fluctuation changes of its value. It focuses on the stage of development and change of a certain subject term itself, and demonstrates the prominence of hot topics. Combining the burst word detection method with the frequency word analysis method can analyze the research hotspots in a certain field.

## 3 Results

### 3.1 The number of literature and general characteristics

**Figure 1:**
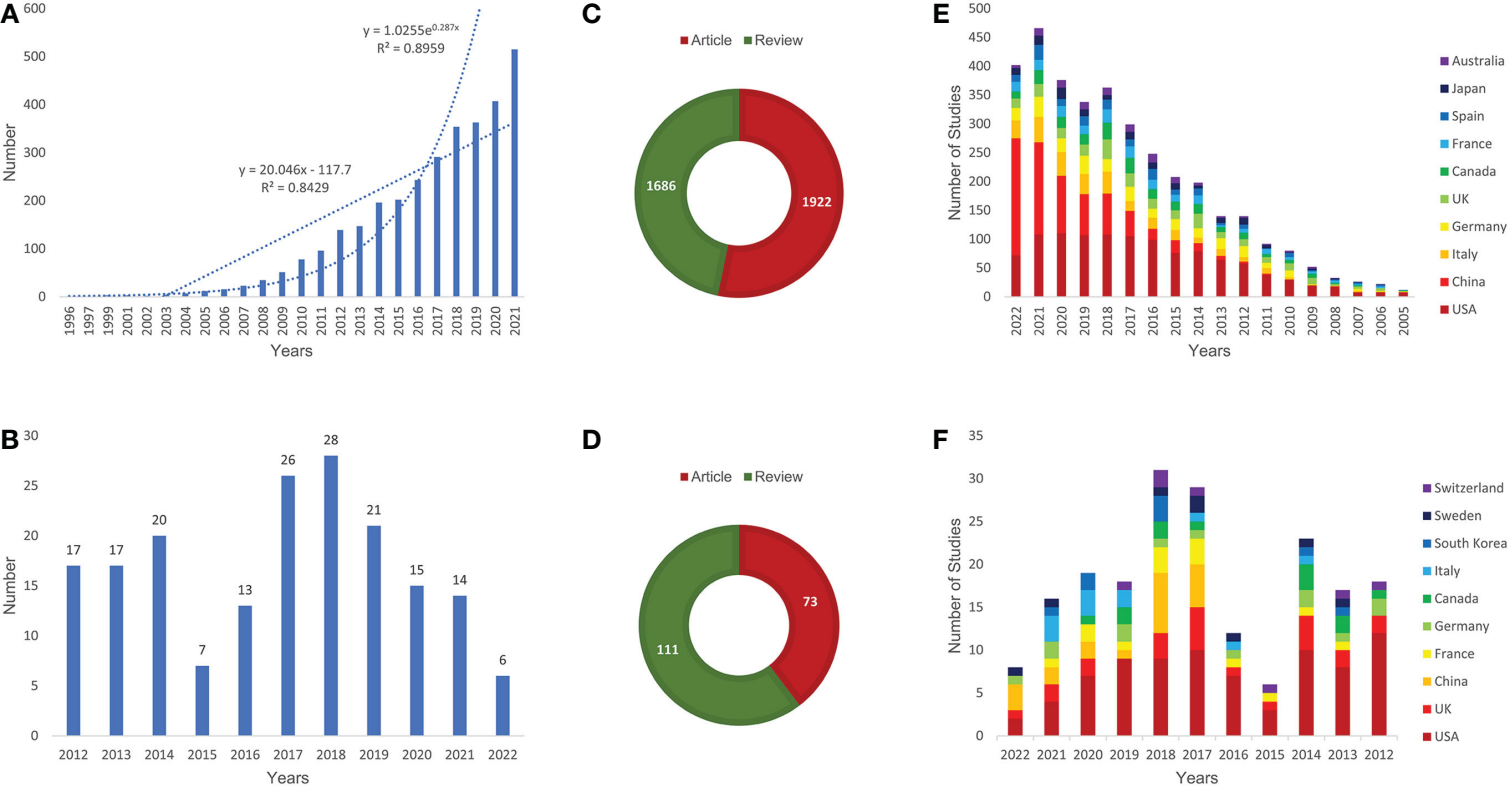
Annual number of publications **(A, B)**, the type of literature **(C, D)**, and the number of publications per year in common countries **(E, F)** of relevant literature. (**A, C, E**: all relevant literature; **B, D, F**: citation classics).

The study found an overall upward trend in the number of publications, indicating that the research on the interaction between microbiota and immune in intestinal inflammatory diseases is gaining attention and is of increasing value for excavation (**Figure 1A**). Currently, the number of literatures published in 2021 had reached the maximum, with 515 articles. The slight decrease in the 419 articles in 2022 might be because the statistics in this study ended on October 12, which was approximately eighty days before the end of 2022, and it was believed that a new peak will be reached after the end of this year. Price’s law is one of the important results of bibliometric law research, describing the changing pattern of a certain type of literature volume over time and predicting the future change trend. According to Price’s law, the growth of literature production was exponential, and the exponential curve equation was y=1.0255e^0.287x^. The simulation curve fits well with the annual literature growth trend with a high coefficient of determination (R^2 =^ 0.8959) (**Figure 1A**). According to the exponential curve equation, the average annual growth rate was 28.7%. By the fitting curve, we can predict that the annual articles will continue to grow in the coming years. In addition, we selected 184 highly cited papers in this field, and referred to these as “citation classics” (**Supplementary Table 1**). Among them, the number of articles published in 2018 was the most, reaching 28 (**Figure 1B**). Most of the document types were articles in 3608 papers (**Figure 1C**), but most of the types were reviews in citation classics (**Figure 1D**). In addition, based on OALM, the number of published articles in common countries was counted year by year (**Figures 1E, F**).

### 3.2 Author analysis

**Figure 2:**
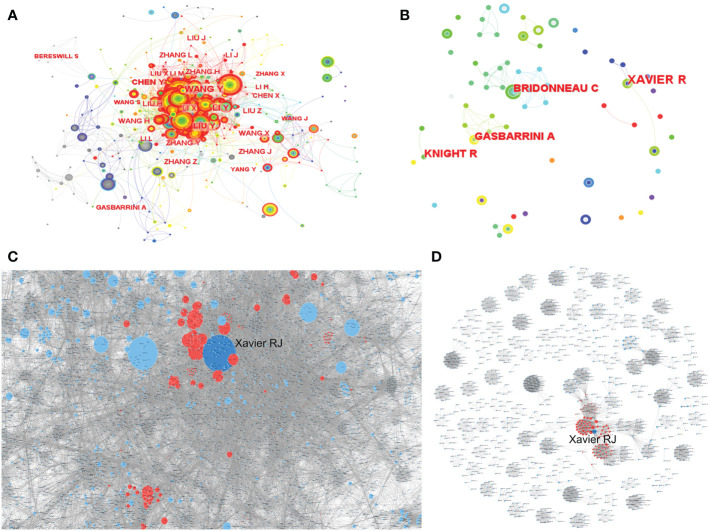
The co-occurrence of authors **(A, B)** and the cooperation relationship between authors **(C, D)** of relevant literature. (**A, C**: all relevant literature; **B, D**: citation classics; **C, D**: Each small blue dot represents an author, the link represents collaboration, and the larger the blue dot, the more collaboration. The dark blue dots are the authors who collaborated the most, and the red dots are the authors who collaborated with that author).

Based on WoSCC, there were 16,364 authors in all relevant research and 1,155 authors in citation classics. Gasbarrini A and Sokol H were the authors with the most cumulative number of articles, with 25 articles, and Xavier RJ was the author with the largest number of publications in citation classics, reaching 7 (**Supplementary Table 2**). Based on CiteSpace, there were 788 authors in all relevant research (**Figure 2A**) and 291 authors in citation classics (**Figure 2B**). Among them, the author with the highest cumulative number of articles was WANG Y, with 71 articles, and Xavier RJ was the author with the largest number of publications in citation classics, reaching 4 (**Supplementary Table 3**). The author collaboration network diagram revealed the existence of collaborative relationships between high-producing authors and the formation of stable research teams, with Xavier RJ being the most cooperative and widely collaborating author in both all studies (**Figure 2C**) and citation classics (**Figure 2D**), implying that Xavier RJ from Massachusetts Institute of Technology was the most influential author in the field.

### 3.3 Institution analysis

**Figure 3:**
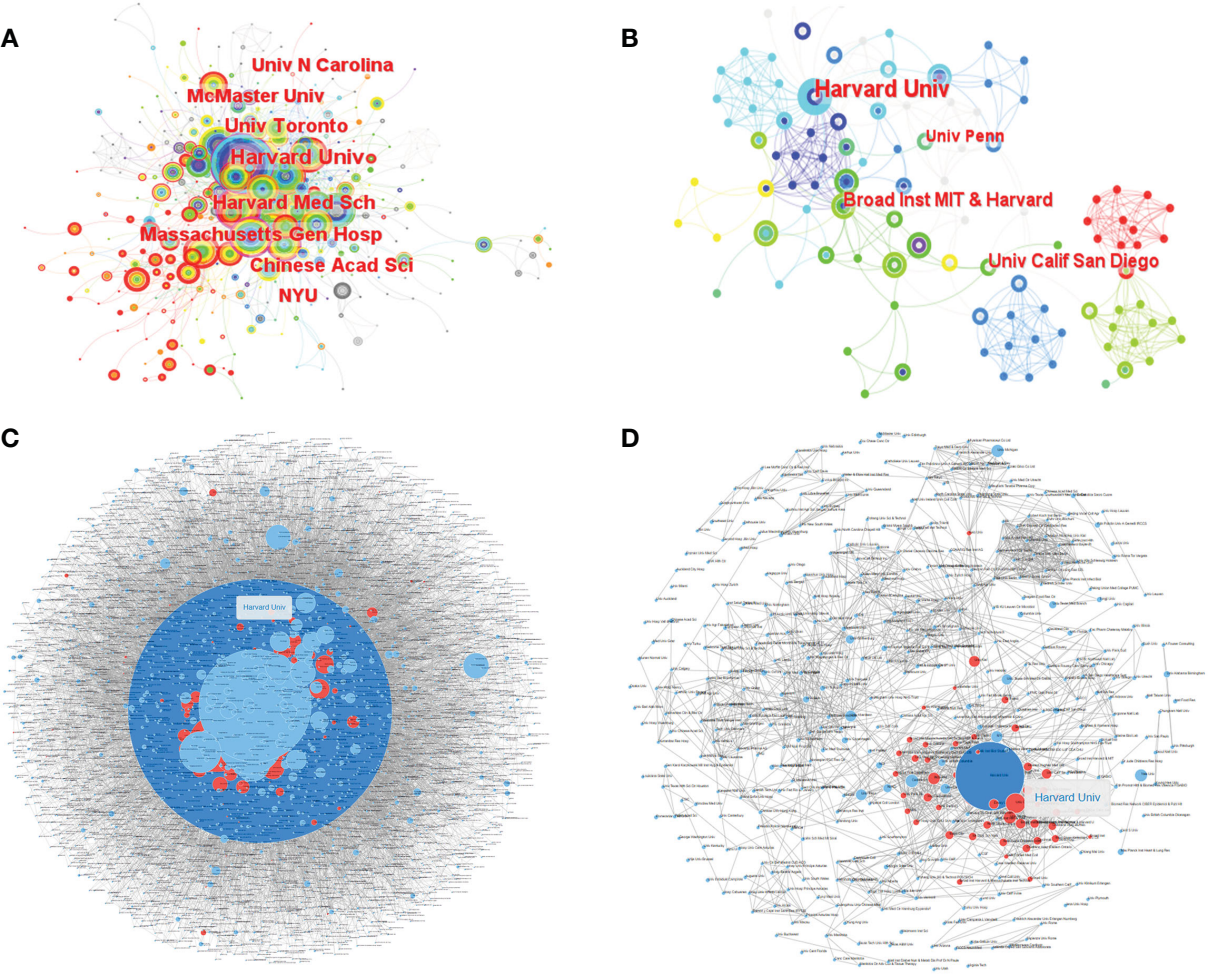
The co-occurrence of institutions **(A, B)** and the cooperation relationship between institutions **(C, D)** of relevant literature. (**A, C**: all relevant literature; **B, D**: citation classics; **C, D**: Each small blue dot represents an institution, the link represents collaboration, and the larger the blue dot, the more collaboration. The dark blue dot is the institution that cooperates the most, and the red dots are the institutions that cooperates with that institution.).

Based on WoSCC, there were 3,248 institutions in all relevant research and 462 institutions in citation classics. Among them, Harvard University had the most articles, reaching 142 articles, and the University of California System had the most articles in citation classics with 17 articles (**Supplementary Table 2**). Based on CiteSpace, there were 502 institutions in all relevant research (**Figure 3A**) and 270 institutions in citation classics (**Figure 3B**). Among them, Harvard University had the most articles, reaching 63 articles, and Harvard University had the most articles in citation classics with 9 articles (**Supplementary Table 4**). The institution collaboration network diagram revealed close collaboration among research institutions, dominated by universities, with Harvard University being the most collaborative and extensive institution in both all studies (**Figure 3C**) and citation classics (**Figure 3D**), indicating that Harvard University had made the most prominent contribution to the field.

### 3.4 Countries analysis

**Figure 4:**
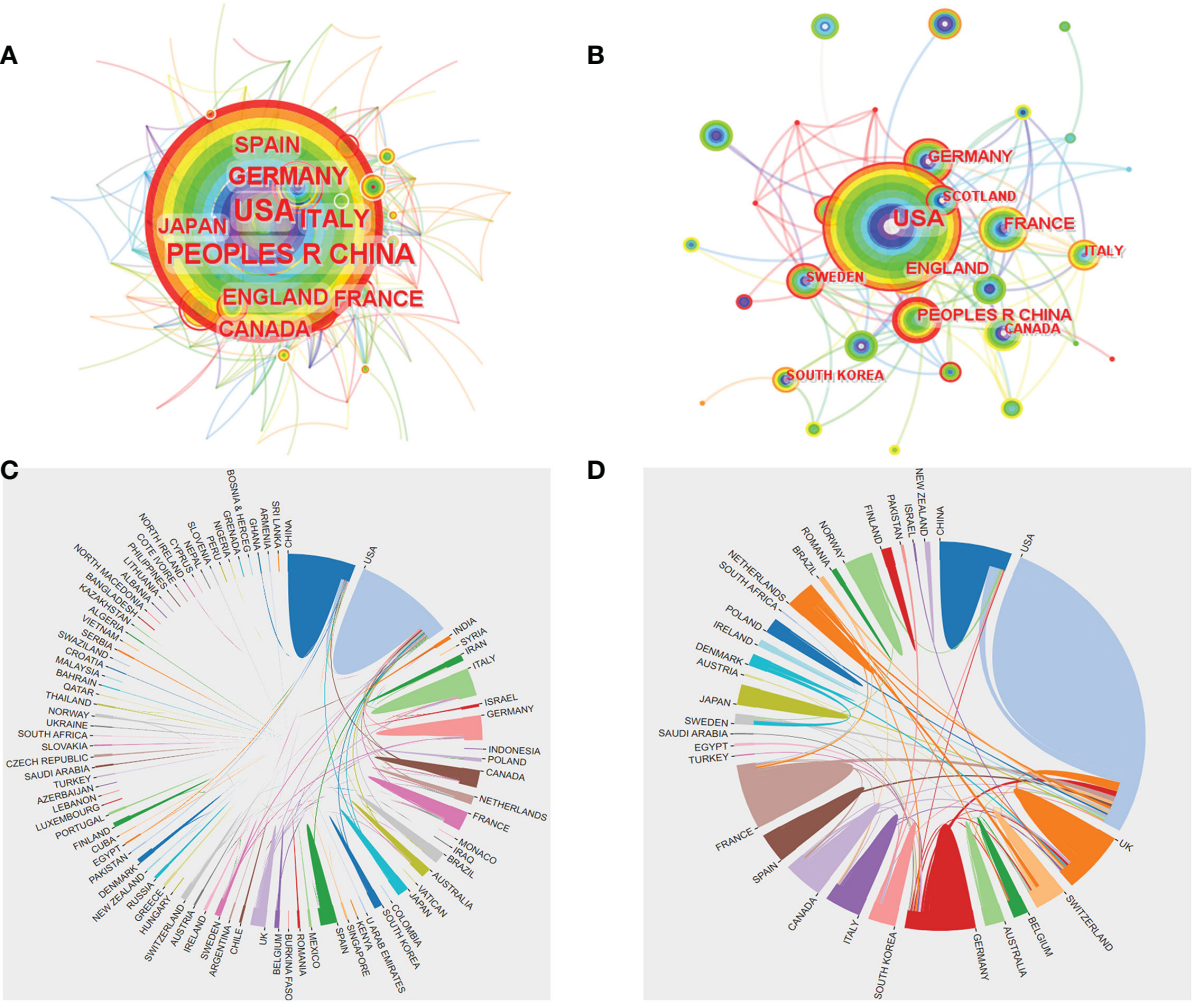
The co-occurrence of countries **(A, B)** and the cooperation relationship between countries **(C, D)** of relevant literature. (**A, C**: all relevant literature; **B, D**: citation classics).

Based on WoSCC, there were 93 countries/regions in all relevant research and 38 countries/regions in citation classics. The USA published the most articles, reaching 1131 articles, and the USA published the most articles with 81 articles in citation classics (**Supplementary Table 2**). Based on CiteSpace, there were 92 countries in all relevant research (**Figure 4A**) and 38 countries in citation classics (**Figure 4B**). Among them, the USA had the most published articles with 1119 articles, followed by CHINA, ITALY, GERMANY, ENGLAND, and CANADA, all with more than 200 articles (**Supplementary Table 5**). Among citation classics, the USA was far ahead, with 81 publications. The country cooperation network map revealed close and stable cooperation among highly productive countries, with the USA cooperating most extensively and far ahead, and half of the countries cooperating less (**Figures 4C, D**).

### 3.5 Journals and cited articles analysis

**Figure 5:**
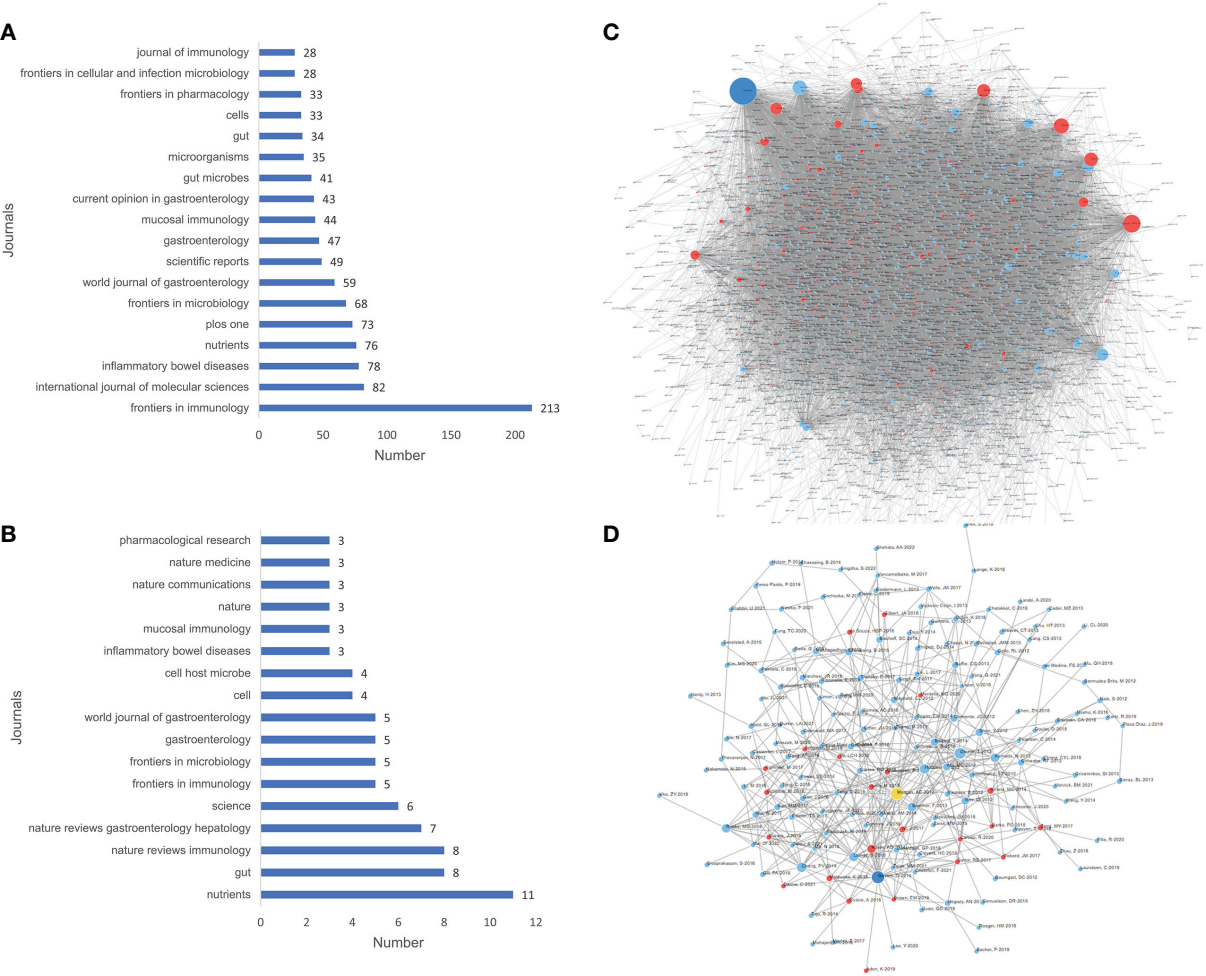
The number of articles published by journals **(A, B)** and the article citation relationship network **(C, D)** of relevant literature. (**A, C**: all relevant literature; **B, D**: citation classics; **C, D:** Each small blue dot represents an article, the links represent citations, and the larger the blue dot, the more citations. The dark blue dot is the most cited article, and the red dots are the articles that cite that article).

Based on WoSCC, related research had been published in 963 journals. Frontiers in Immunology (2021 IF=8.787; Q1) contributed the most to this research field, reaching 213 articles (**Figure 5A**). Among the citation classics, 102 journals were involved, and Nutrients (2021 IF= 6.706; Q1) published the most articles, reaching 11 articles (**Figure 5B**). The ranking of citation times of journals was shown in **Supplementary Table 6**. The article (Round and Mazmanian, 2009) was the most cited, with 2946 citations and an average of 210 citations per year, Among the citation classics, the article(Hooper et al., 2012) was cited the most, with 2429 citations and an average of 220 citations per year. The article citation relationship network diagram showed that the mutual references were relatively close (**Figures 5C, D**). The article (Frank et al., 2007) was the most cited among these 3608 articles cross-cited (**Figure 5C**), and the article (Gevers et al., 2014) was the most cited among these 184 citation classics cross-cited (**Figure 5D**).

### 3.6 Keywords analysis

#### 3.6.1 Word frequency analysis

The keywords reflect the core content of the research in a concise form, and the analysis of high-frequency keywords can accurately reveal the research hotspots and overall development trends of the field. The keywords counted in the studied literature reflect some extent the importance of the keywords and can be useful for detecting the evolution of research hotspots (Sun et al., 2012). Through the analysis of keywords, this study also presented keywords that reflected intestinal related diseases, such as “inflammatory bowel disease”, “Crohn’s disease”, “ulcerative colitis”, “irritable bowel syndrome”, “colorectal cancer” etc.; reflected gut microbiota, such as “gut microbiota”, “intestinal microbiota”, “bacteria”, “fecal microbiota”, “commensal microbiota” etc.; reflected immune system, such as “immune system”, “immune response”, “regulatory T cell”, “dendritic cell”, “innate lymphoid cell”, “innate immunity” etc.; reflected type of bacteria, such as “commensal bacteria”, “clostridium difficile infection”, “Escherichia coli”, “segmented filamentous bacteria”, “Faecalis bacterium prausnitzii”, “lactic acid bacteria”, “akkermansia muciniphila” etc.; reflected mechanism of intestinal inflammatory diseases, such as “mucosal immunity”, “genome wide association”, “hygiene hypothesis”, “chain fatty acid”, “activation”, “aryl hydrocarbon receptor”, “gene expression”, “oxidative stress”, “gene expression”, “barrier function” etc. (**Table 1**).

**Table 1:** Top 15 keywords of relevant literature.

Category	Rank	Keywords	Frequency	Keywords	Centrality	Keywords	Degree	Keywords	Σ
A. All related researches	1	inflammatory bowel disease	1277	Escherichia coli	0.06	Crohn’s disease	90	Crohn’s disease	2.9
	2	gut microbiota	1136	Crohn’s disease	0.05	Escherichia coli	87	toll like receptor	2.02
	3	Crohn’s disease	745	gastrointestinal tract	0.05	experimental colitis	85	dendritic cell	1.67
	4	ulcerative colitis	695	disease	0.05	activation	84	Escherichia coli	1.35
	5	intestinal microbiota	596	intestinal epithelial cell	0.05	bacteria	82	segmented filamentous bacteria	1.33
	6	microbiota	311	experimental colitis	0.04	chain fatty acid	80	flora	1.29
	7	regulatory T cell	304	activation	0.04	expression	80	genome wide association	1.27
	8	chain fatty acid	291	bacteria	0.04	dendritic cell	78	experimental colitis	1.24
	9	T cell	280	chain fatty acid	0.04	colitis	77	regulatory T cell	1.24
	10	expression	279	dendritic cell	0.04	colonization	77	placebo controlled trial	1.23
	11	immune response	266	lactic acid bacteria	0.04	fecal microbiota	76	intestinal epithelial cell	1.22
	12	inflammation	265	regulatory T cell	0.03	regulatory T cell	75	Faecalis bacterium prausnitzii	1.18
	13	dendritic cell	256	ulcerative colitis	0.03	invasive Escherichia coli	73	invasive Escherichia coli	1.15
	14	bacteria	251	mucosal immunity	0.03	ulcerative colitis	72	hygiene hypothesis	1.14
	15	disease	247	fecal microbiota	0.03	gastrointestinal tract	72	commensal bacteria	1.14
B. Citation classics	1	inflammatory bowel disease	68	inflammatory bowel disease	0.36	inflammatory bowel disease	79	gut microbiota	2.34
	2	gut microbiota	53	gut microbiota	0.34	gut microbiota	78	ulcerative colitis	1.37
	3	Crohn’s disease	45	intestinal microbiota	0.28	intestinal microbiota	76	toll like receptor	1.24
	4	intestinal microbiota	43	Crohn’s disease	0.21	Crohn’s disease	69	chain fatty acid	1.21
	5	regulatory T cell	29	regulatory T cell	0.18	regulatory T cell	55	dendritic cell	1.08
	6	chain fatty acid	27	ulcerative colitis	0.15	ulcerative colitis	49	immune system	1.08
	7	ulcerative colitis	26	toll like receptor	0.1	fecal microbiota	40	T cell	1.07
	8	toll like receptor	15	colorectal cancer	0.1	chain fatty acid	39	segmented filamentous bacteria	1.05
	9	fecal microbiota	15	bacteria	0.09	toll like receptor	39	intestinal epithelial cell	1.04
	10	aryl hydrocarbon receptor	12	double blind	0.09	innate lymphoid cell	34	microbiota	1.04
	11	innate lymphoid cell	12	chain fatty acid	0.08	colorectal cancer	34	commensal microbiota	1.03
	12	colorectal cancer	10	fecal microbiota	0.07	aryl hydrocarbon receptor	32	mice	1.03
	13	T cell	9	innate lymphoid cell	0.07	akkermansia muciniphila	32	intestinal microbiota	1.03
	14	irritable bowel syndrome	9	aryl hydrocarbon receptor	0.06	bacteria	27	response	1.02
	15	clostridium difficile infection	8	dietary fiber	0.06	clostridium difficile infection	27	diet induced obesity	1.02

**Figure 6:**
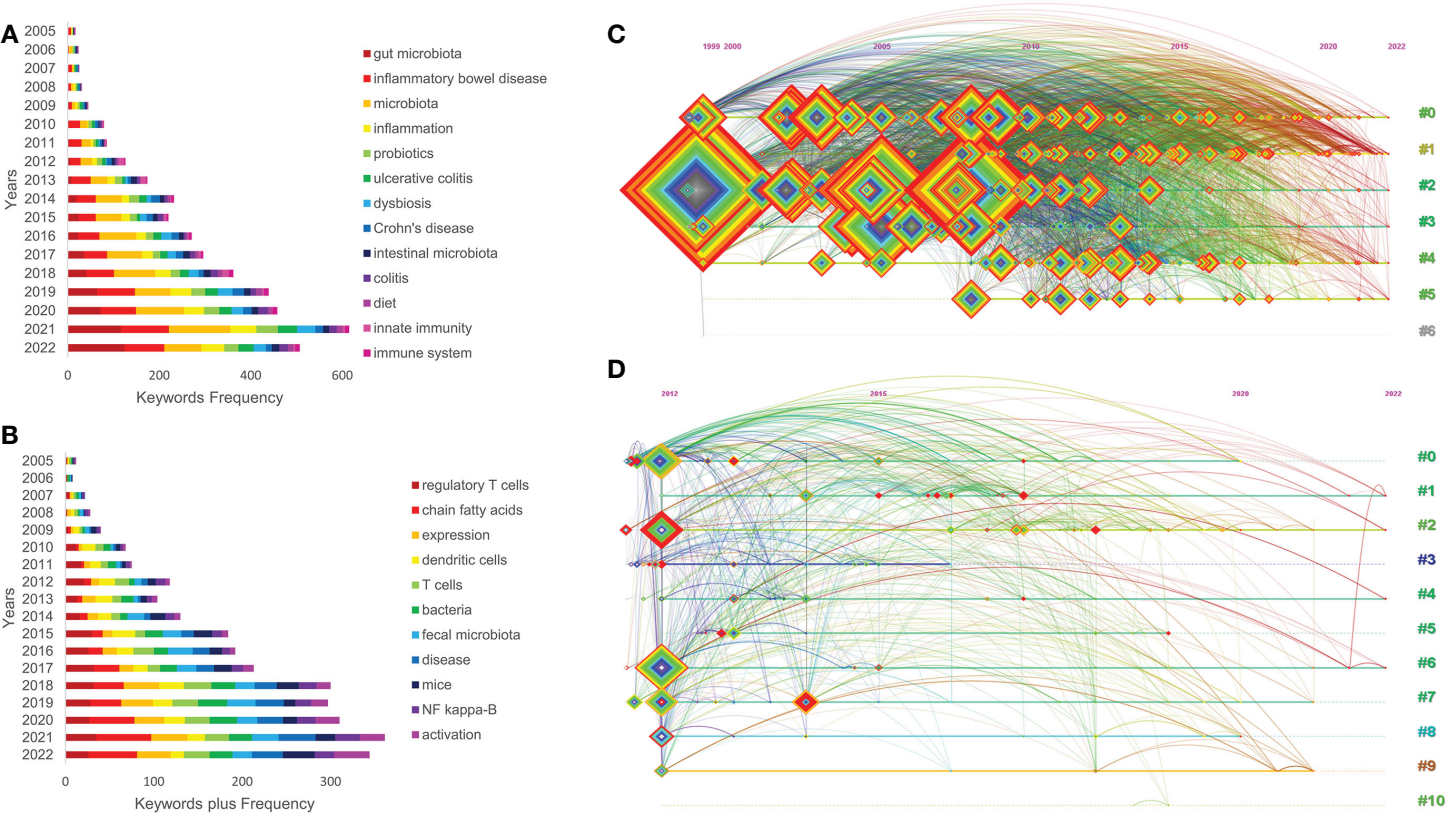
Annual number of common keywords **(A, B)**, the clustering timeline view of keywords **(C, D)** of relevant literature. (**A, B, C**: all relevant literature; **D**: citation classics).

There were 770 keywords obtained in all related research, of which there were 5 keywords with word frequency ≥500, and there were 301 keywords obtained in citation classics, 7 of which have a word frequency ≥20. The influential keywords included inflammatory bowel disease (degree: 79; centrality: 0.36), gut microbiota (degree: 78; centrality: 0.34), intestinal microbiota (degree: 76; centrality: 0.28), Crohn’s disease (degree: 69; centrality: 0.21), regulatory T cell (degree: 55; centrality: 0.18), ulcerative colitis (degree: 49; centrality: 0.15), toll like receptor (degree: 40; centrality: 0.1), colorectal cancer (degree: 39; centrality: 0.1) (**Table 1**). In addition, this study also visualized the evolution of common keywords year by year (**Figures 6A, B**).

#### 3.6.2 Cluster analysis

The scattered subject terms are classified and the co-word matrix is manipulated using clustering statistics to cluster subject terms with high co-occurrence frequency into small clusters that are displayed on the timeline to explore potential information. In bibliometric clustering, a cluster often reflects the research theme and focus of a discipline. In this study, CiteSpace software was used to perform cluster analysis on title words and keywords respectively, and the clusters were labeled using the log-likelihood ratio (LLR) method to obtain the clustering timeline views of all related research (**Figure 6C**) and citation classics (**Figure 6D**). The larger the LLR of a word, the more representative the word is for this cluster. There are two important indicators for evaluating the effectiveness of cluster mapping, namely Modularity Q (Q, value interval [0,1]) and Weighted Mean Silhouette S (S, value interval [-1,1]). The Q value is used to assess the performance of the clustered network, the higher the value, the better the construction of the clustered network. The S value is used to measure the homogeneity of the cluster members, and the larger the value, the better the consistency among the class members. Q>0.3 and S>0.5 represent that the obtained clustering network structures are obviously convinced and the clustering outcomes are rational, respectively. The Q values of 0.3042 and 0.4804 (both>0.3) and S values of 0.6406 and 0.7696 (both>0.6) in **Figures 6C, D** indicated that these clustering maps were reasonable and informative.

In all related research, 7 meaningful clusters were formed (**Table 2**), and 11 clusters were formed in citation classics (**Table 2**). From #0 to #10, the smaller the number, the more keywords were included in the cluster. There were multiple overlapping clusters in the clustering diagram, indicating that they were strongly correlated. The clustering timeline view visualized the period of each cluster and the association between different clusters, which displaying the evolution of the research. The cluster analysis in this study revealed that the most researched content was related to IBD, while the other three areas were mainly focused on: ①research on mechanisms of intestinal inflammatory diseases, such as anti-inflammatory properties, gut-brain axis, b-cell system and activation; ②research on IBD related diseases, such as colorectal cancer and IBD-associated CRC; ③research on the treatment of intestinal inflammatory diseases, such as fecal microbiota transplantation and potential novel therapeutics; ④research on intestinal statuses, such as intestinal microbiota, intestinal diversity, gut microbiota, host development, gut dysbiosis and intestinal epithelium.

**Table 2:** Keywords co-occurrence network clustering table.

Category	Cluster ID	Size	Mean (Year)	Top terms (top 5)
A. All related researches—Title words cluster	0#	173	2011	anti-inflammatory properties (2870.68, 1.0E-4); gram-positive cell wall (2615.23, 1.0E-4); normal intestinal microbiota (2615.23, 1.0E-4); regulatory t cell (2581.43, 1.0E-4); myeloid cell (2418.3, 1.0E-4)
	1#	170	2016	gut-brain axis (6627.4, 1.0E-4); multiple sclerosis (3641.4, 1.0E-4); fatty acid (2692.22, 1.0E-4); inflammatory bowel disease (2500.25, 1.0E-4); diabetes mellitus (2190.1, 1.0E-4)
	2#	156	2009	inflammatory bowel disease (11369.38, 1.0E-4); intestinal inflammation (5113.07, 1.0E-4); gastrointestinal tract (3821.67, 1.0E-4); commensal microbiota (3141.74, 1.0E-4); toll-like receptor (3071.84, 1.0E-4)
	3#	119	2009	fecal microbiota transplantation (2764.49, 1.0E-4); intestinal inflammation (2722.06, 1.0E-4); human microbiome (2560.7, 1.0E-4); health benefit (2449.09, 1.0E-4); scientific evidence (2324.55, 1.0E-4)
	4#	112	2015	inflammatory bowel disease (5082.06, 1.0E-4); gut microbiome (4083.55, 1.0E-4); juvenile idiopathic arthritis (3623.68, 1.0E-4); narrative review (2762.35, 1.0E-4); pediatric inflammatory bowel diseases (2476.82, 1.0E-4)
	5#	32	2015	colorectal cancer (3832.71, 1.0E-4); colorectal tumorigenesis (1109.32, 1.0E-4); indirect effect (902.54, 1.0E-4); additive titanium dioxide (879.63, 1.0E-4); inflammatory pathogenesis (871.96, 1.0E-4)
	6#	8	1999	b-cell system (25.29, 1.0E-4); human mucosae (25.29, 1.0E-4); exocrine gland (25.29, 1.0E-4); inflammatory bowel disease (0.5, 0.5); gut microbiota (0.44, 1.0)
B. All related researches—Keywords cluster	0#	173	2011	activation (73.91, 1.0E-4); expression (68.89, 1.0E-4); cell (65.77, 1.0E-4); colitis (54.89, 1.0E-4); mice (51.82, 1.0E-4)
	1#	170	2016	inflammatory bowel disease (55.95, 1.0E-4); gut-brain axis (55.63, 1.0E-4); neuroinflammation (39.55, 1.0E-4); obesity (34.67, 1.0E-4); Crohn’s disease (33.96, 1.0E-4)
	2#	156	2009	inflammatory bowel disease (96.46, 1.0E-4); Crohn’s disease (54.22, 1.0E-4); genome wide association (49.54, 1.0E-4); commensal bacteria (41.37, 1.0E-4); ulcerative colitis (40.43, 1.0E-4)
	3#	119	2009	intestinal microbiota (40.05, 1.0E-4); probiotics (37.34, 1.0E-4); fecal microbiota transplantation (36.82, 1.0E-4); irritable bowel syndrome (34.15, 1.0E-4); lactic acid bacteria (31.07, 1.0E-4)
	4#	112	2015	diversity (31.71, 1.0E-4); intestinal microbiome (25.48, 1.0E-4); ankylosing spondylitis (23.94, 1.0E-4); diet (21.92, 1.0E-4); colorectal cancer (21.57, 1.0E-4)
	5#	32	2015	colorectal cancer (104.07, 1.0E-4); colon cancer (44.06, 1.0E-4); stem cell (19.98, 1.0E-4); colitis-associated cancer (19.5, 1.0E-4); endoplasmic reticulum stress (17.01, 1.0E-4)
	6#	8	1999	Epstein Barr virus (14.04, 0.001); peripheral lymph node (14.04, 0.001); j chain gene (14.04, 0.001); follicular dendritic cell (14.04, 0.001); IgA deficient patient (14.04, 0.001)
C. Citation classics—Title words cluster	0#	56	2015	inflammatory disease (91.68, 1.0E-4); gut microbiota (67.81, 1.0E-4); butyrate-producing colon bacteria (59.81, 1.0E-4); human gut (59.81, 1.0E-4); pathogen colonization (56.93, 1.0E-4)
	1#	39	2016	short chain (81.1, 1.0E-4); microbiota metabolite (52.43, 1.0E-4); fatty acids GPCR (52.43, 1.0E-4); distant organ (48.36, 1.0E-4); epithelial barrier (44.29, 1.0E-4)
	2#	38	2019	gut microbiota (65.07, 1.0E-4); changing ecosystem (42.98, 1.0E-4); age environment diet (42.98, 1.0E-4); intestinal permeability (42.81, 1.0E-4); inflammatory bowel disease (41.82, 1.0E-4)
	3#	30	2013	intestinal tract (54.55, 1.0E-4); bacterial diversity (54.55, 1.0E-4); following allogeneic hematopoietic stem cell transplantation (54.55, 1.0E-4); allergic inflammation (48.41, 1.0E-4); commensal fungi (42.29, 1.0E-4)
	4#	26	2016	potential novel therapeutics (50.89, 1.0E-4); human health (44.17, 1.0E-4); basic science (36.17, 1.0E-4); early life (34.62, 1.0E-4); lactobacillus reuteri (28.87, 1.0E-4)
	5#	26	2015	host development (56.91, 1.0E-4); risk pathogenesis prevention (49.68, 1.0E-4); diverging inflammasome signal (28.19, 1.0E-4); human health (21.88, 1.0E-4); gut microbiota (17.64, 1.0E-4)
	6#	26	2016	natural polysaccharide (56.58, 1.0E-4); programming health (48.56, 1.0E-4); potential role (48.56, 1.0E-4); phytogenic substance (45.06, 1.0E-4); cesarean section (41.58, 1.0E-4)
	7#	25	2015	colorectal cancer (67, 1.0E-4); central mediator (56.53, 1.0E-4); gut-brain communication (56.53, 1.0E-4); colitis-associated neoplasia (52.17, 1.0E-4); gut microbiota composition (47.83, 1.0E-4)
	8#	15	2016	brain-gut interaction (47.28, 1.0E-4); functional gastrointestinal disorder (33.8, 1.0E-4); irritable bowel syndrome (33.8, 1.0E-4); inflammatory bowel disease (32.82, 1.0E-4); host-microbiota interaction (27.23, 1.0E-4)
	9#	13	2019	related diseases (38.56, 1.0E-4); rheumatoid arthritis (28.74, 1.0E-4); gut-joint axis (28.74, 1.0E-4); Mediterranean diet (19.05, 1.0E-4); IBD patient (19.05, 1.0E-4)
	10#	4	2019	candida albican (13.48, 0.001); pathology (13.48, 0.001); cross-reactivity (13.48, 0.001); gut microbiota (0.39, 1.0); intestinal microbiota (0.3, 1.0)
D. Citation classics—Keywords cluster	0#	56	2015	gut dysbiosis (145.24, 1.0E-4); symbiotic bacteria (141.8, 1.0E-4); Gi tract (126.96, 1.0E-4); butyrate-producing colon bacteria (119.07, 1.0E-4); butyrogenic effect (119.07, 1.0E-4)
	1#	39	2016	intestinal epithelium (167.38, 1.0E-4); intestinal homeostasis (132.59, 1.0E-4); bacterial metabolite (94.25, 1.0E-4); gut bacteria (82.44, 1.0E-4); crucial role (75.64, 1.0E-4)
	2#	38	2019	neurofibrillary tangle (164.23, 1.0E-4); beta peptide (138.47, 1.0E-4); systemic inflammation (99.91, 1.0E-4); gut microbiota balance (99.08, 1.0E-4); external factor (78.56, 1.0E-4)
	3#	30	2013	intestinal diversity (115.43, 1.0E-4); t-reg cell (76.88, 1.0E-4); mucosal interface (76.88, 1.0E-4); allergic asthma (69.02, 1.0E-4); pulmonary infection (64.05, 1.0E-4)
	4#	26	2016	early-life gut microbiota (78.78, 1.0E-4); intestinal permeability (56.2, 1.0E-4); microbial translocation (52.49, 1.0E-4); axis-related dysfunction (45.92, 1.0E-4); neuroactive metabolite (45.92, 1.0E-4)
	5#	26	2015	IBD-associated CRC (188.14, 1.0E-4); bacterial microbiota (66.98, 1.0E-4); smoking cessation (66.98, 1.0E-4); oxidative stress (66.98, 1.0E-4); homeostatic regulation (53.57, 1.0E-4)
	6#	26	2016	cesarean delivery (277.36, 1.0E-4); natural polysaccharide (257.09, 1.0E-4); anti-inflammatory effect (155.85, 1.0E-4); acute colitis (130.75, 1.0E-4); phytogenic substance (98.94, 1.0E-4)
	7#	25	2015	colorectal cancer (138.98, 1.0E-4); molecular mechanism (119.41, 1.0E-4); various mechanism (107.04, 1.0E-4); normal mice (107.04, 1.0E-4); Japanese colorectal cancer (90.54, 1.0E-4)
	8#	15	2016	brain-gut axis (96.26, 1.0E-4); risk factor (60.11, 1.0E-4); disease course (48.08, 1.0E-4); salivary cortisol (48.08, 1.0E-4); pharmacologic nutritional (48.08, 1.0E-4)
	9#	13	2019	related diseases (197.89, 1.0E-4); intestinal barrier disruption (98.58, 1.0E-4); metabolic disorder (83.96, 1.0E-4); intestinal bacteria (73.8, 1.0E-4); diabetes patient (65.64, 1.0E-4)
	10#	4	2019	th17 cell (26.35, 1.0E-4); albican (26.35, 1.0E-4); cross-reactivity (13.14, 0.001); protection (13.14, 0.001); single ubiquitous member (13.14, 0.001)

#### 3.6.3 Emergent analysis

Keywords emergence refers to the degree of the sudden change in the frequency of a keyword co-occurrence within a period, and is mainly used to study the hotspots in a specific period. The emerging words in the literature on the interaction between microbiota and immune in intestinal inflammatory diseases were analyzed by CiteSpace software. Each emergence word has a red highlighted bar consisting of each cell and a blue bar for the rest of the cells, where each cell represents a year (**Table 3**). In the early period of 1997-2022, research points were mainly genomics such as 16S ribosomal RNA and genome wide association, and growth in children, such as body weight indicators and dietary nutritional intake. In the midterm period, research points were mainly intestinal bacterial infections such as fecal microbiota and commensal bacteria, and immune system research, such as regulatory T cells and toll-like receptors. In the later period, research points were mainly mechanisms of intestinal inflammatory diseases, such as gut-liver axis, short-chain fatty acid, intestinal inflammation, and gut dysbiosis. It was notable that research on fusobacterium nucleatum, gut-liver axis, and short-chain fatty acid might be a future research trend. The citation classics focused on the period from 2012 to 2022, and emergent analysis showed that the hotspots were mainly in the study of mechanisms related to intestinal microbiota and immune system regulation.

**Table 3:** Emergent analysis of keywords in relevant literature.

Category	Keywords	Strength	Begin	End	1997- 2022
A. All related researches	oral tolerance	7.82	1999	2015	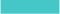 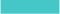             
	dendritic cell	14.61	2004	2013	
	16S ribosomal RNA	6.65	2004	2013	
	Crohn’s disease	22.68	2005	2012	
	hygiene hypothesis	7.52	2005	2016	
	adaptive immunity	8.01	2006	2015	
	toll like receptor	22.92	2007	2014	
	placebo controlled trial	9.75	2007	2015	
	genome wide association	9.75	2008	2012	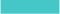 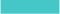 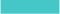 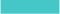 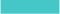 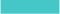 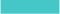 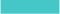 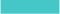 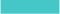 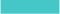      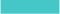 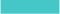 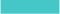 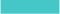 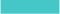 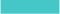 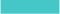 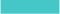 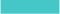 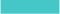
	*in vivo*	8.91	2008	2014	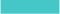 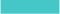 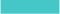 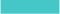 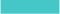 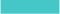 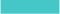 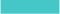 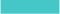 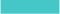 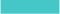        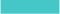 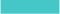 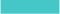 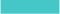 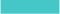 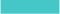 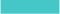 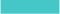
	commensal bacteria	6.44	2009	2015	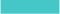 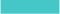 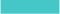 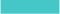 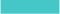 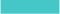 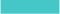 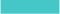 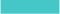 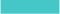 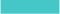 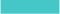        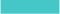 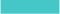 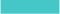 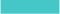 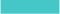 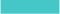 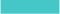
	clostridium difficile	6.29	2009	2016	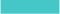 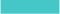 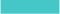 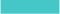 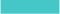 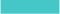 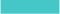 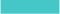 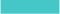 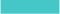 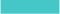 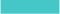         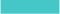 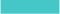 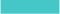 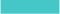 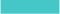 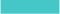
	TGF beta	6.13	2009	2013	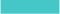 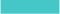 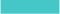 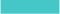 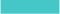 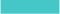 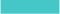 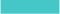 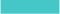 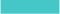 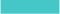 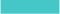      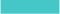 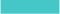 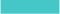 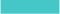 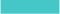 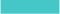 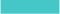 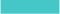 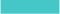
	helicobacter hepaticus	5.77	2009	2015	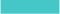 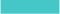 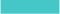 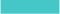 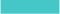 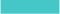 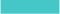 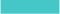 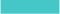 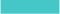 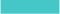 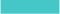        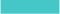 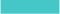 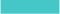 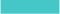 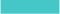 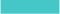 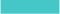
	Paneth cell	5.67	2009	2013	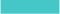 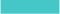 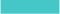 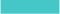 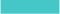 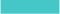 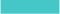 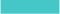 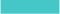 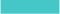 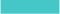 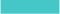      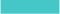 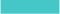 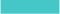 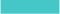 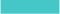 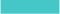 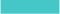 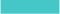 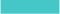
	segmented filamentous bacteria	18.09	2010	2015	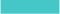 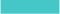 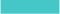 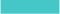 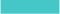 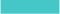 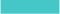 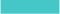 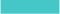 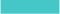 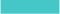 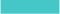 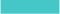       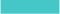 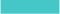 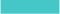 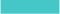 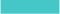 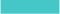 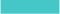
	germ free	7.91	2010	2015	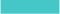 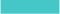 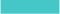 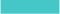 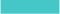 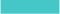 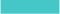 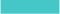 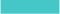 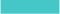 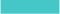 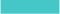 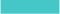       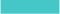 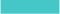 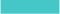 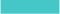 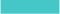 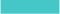 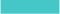
	innate immunity	7.14	2010	2015	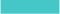 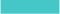 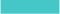 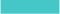 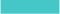 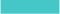 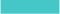 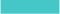 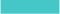 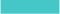 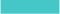 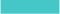 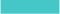       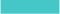 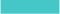 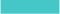 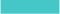 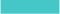 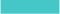 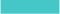
	susceptibility loci	7.02	2011	2012	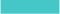 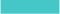 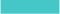 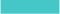 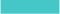 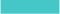 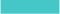 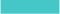 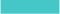 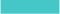 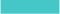 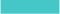 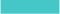 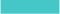   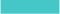 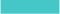 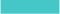 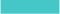 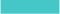 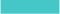 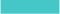 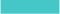 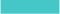 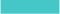
	regulatory T cell	6.64	2011	2012	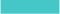 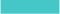 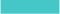 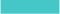 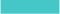 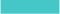 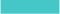 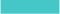 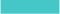 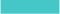 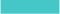 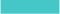 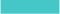 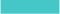   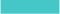 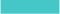 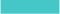 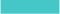 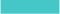 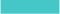 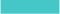 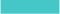 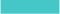 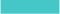
	ROR gamma T	6.45	2011	2015	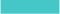 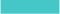 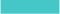 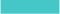 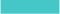 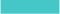 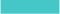 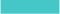 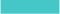 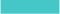 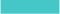 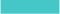 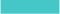 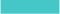      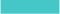 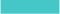 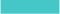 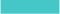 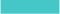 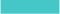 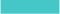
	ileal mucosa	6.11	2011	2016	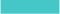 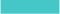 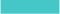 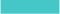 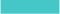 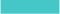 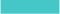 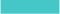 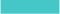 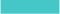 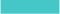 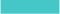 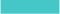 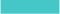       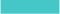 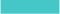 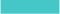 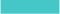 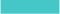 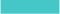
	commensal microbiota	8.88	2012	2017	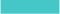 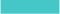 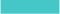 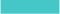 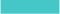 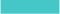 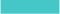 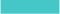 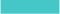 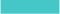 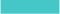 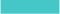 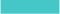 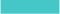 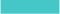       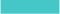 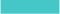 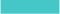 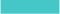 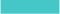
	mucosa associated microbiota	7.41	2012	2017	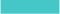 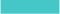 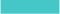 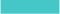 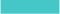 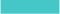 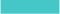 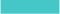 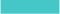 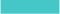 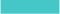 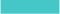 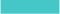 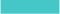 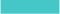       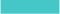 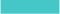 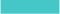 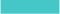 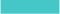
	retinoic acid	6.37	2012	2013	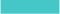 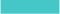 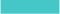 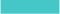 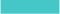 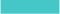 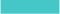 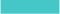 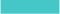 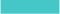 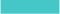 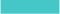 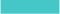 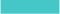 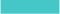   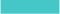 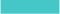 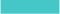 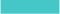 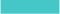 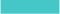 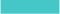 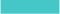 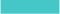
	fecal microbiota	7.22	2014	2017	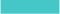 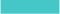 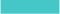 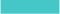 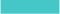 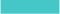 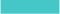 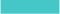 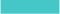 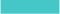 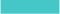 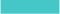 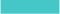 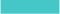 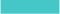 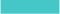 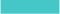     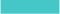 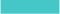 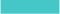 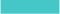 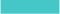
	Faecalis bacterium prausnitzii	6.58	2014	2016	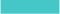 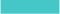 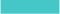 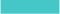 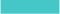 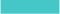 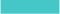 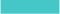 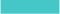 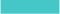 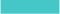 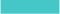 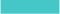 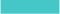 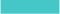 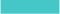 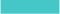    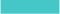 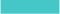 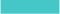 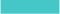 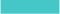 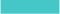
	randomized controlled trial	6.12	2014	2018	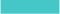 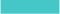 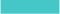 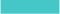 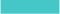 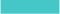 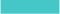 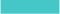 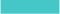 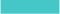 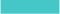 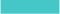 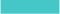 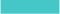 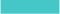 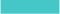 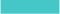      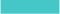 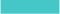 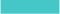 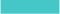
	antibiotic treatment	5.63	2014	2016	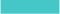 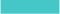 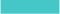 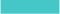 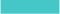 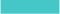 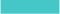 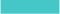 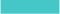 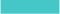 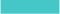 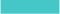 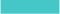 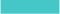 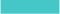 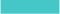 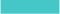    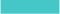 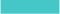 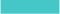 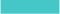 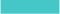 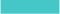
	Commensal bacteria	5.58	2014	2018	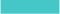 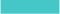 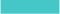 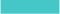 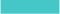 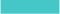 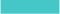 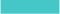 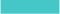 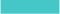 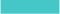 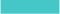 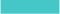 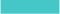 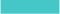 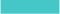 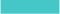      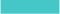 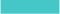 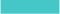 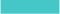
	clostridium difficile infection	7.23	2015	2018	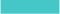 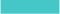 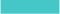 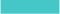 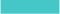 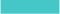 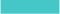 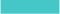 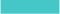 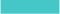 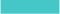 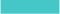 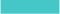 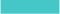 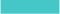 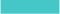 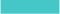 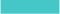     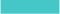 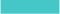 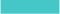 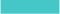
	intestinal inflammation	7.58	2016	2018	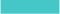 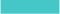 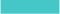 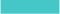 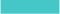 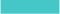 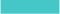 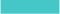 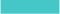 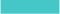 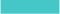 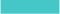 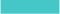 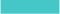 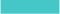 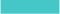 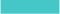 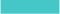 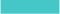    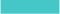 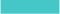 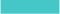 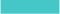
	murine model	6.6	2017	2018	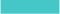 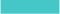 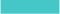 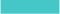 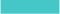 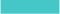 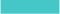 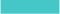 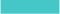 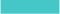 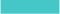 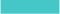 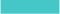 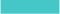 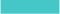 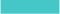 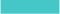 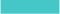 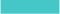 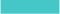   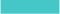 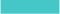 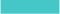 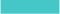
	differentiation	6.91	2019	2020	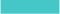 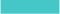 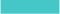 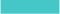 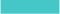 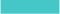 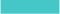 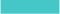 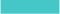 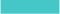 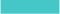 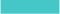 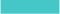 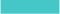 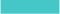 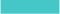 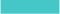 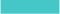 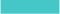 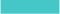 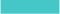 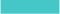   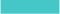 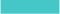
	prebiotics	6.3	2019	2020	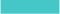 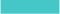 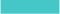 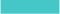 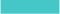 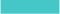 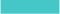 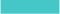 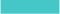 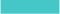 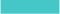 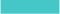 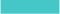 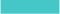 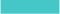 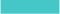 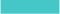 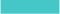 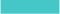 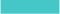 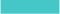 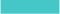   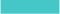 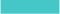
	growth performance	5.8	2019	2022	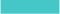 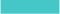 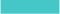 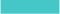 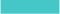 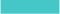 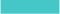 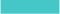 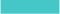 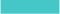 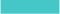 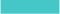 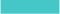 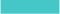 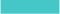 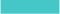 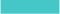 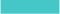 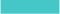 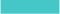 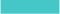 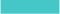    
	short-chain fatty acid	7.52	2020	2022	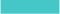 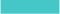 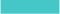 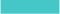 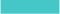 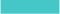 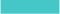 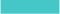 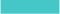 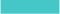 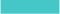 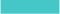 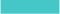 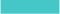 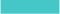 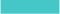 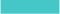 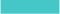 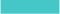 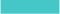 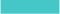 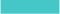 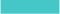   
	bile acid	6.62	2020	2022	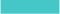 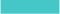 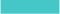 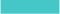 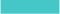 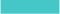 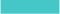 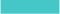 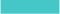 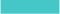 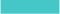 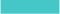 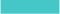 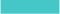 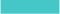 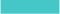 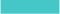 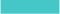 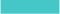 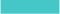 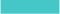 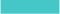 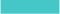   
	gut dysbiosis	6.58	2020	2022	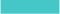 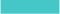 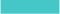 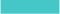 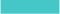 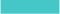 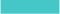 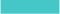 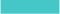 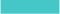 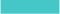 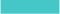 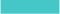 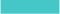 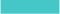 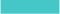 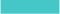 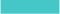 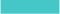 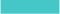 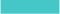 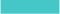 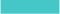   
	gut-liver axis	5.79	2020	2022	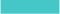 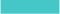 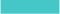 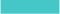 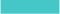 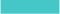 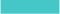 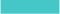 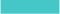 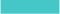 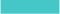 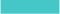 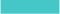 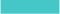 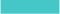 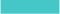 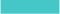 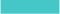 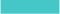 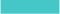 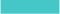 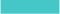 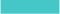   
	fusobacterium nucleatum	5.64	2020	2022	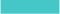 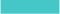 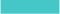 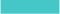 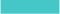 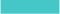 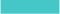 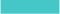 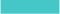 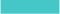 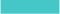 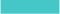 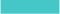 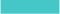 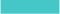 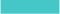 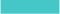 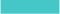 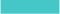 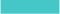 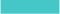 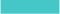 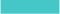   
B. Citation classics	dendritic cell	2.59	2012	2015	    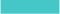 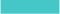 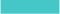 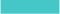 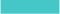 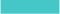 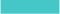 (2012-2022)
	response	2.24	2012	2012	 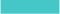 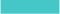 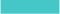 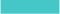 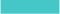 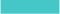 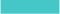 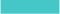 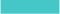 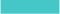 (2012-2022)
	toll like receptor	2.15	2012	2013	  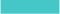 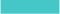 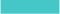 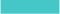 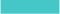 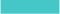 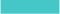 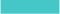 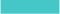 (2012-2022)
	mice	2.08	2012	2014	   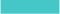 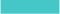 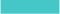 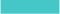 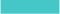 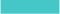 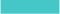 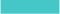 (2012-2022)
	segmented filamentous bacteria	2.02	2012	2013	  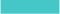 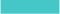 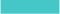 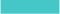 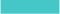 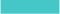 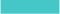 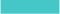 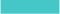 (2012-2022)
	recognition	1.81	2012	2012	 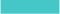 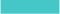 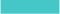 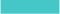 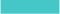 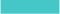 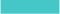 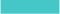 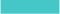 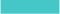 (2012-2022)
	obesity	1.82	2013	2013	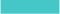  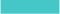 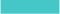 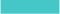 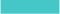 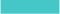 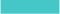 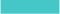 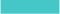 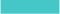 (2012-2022)
	ROR gamma T	1.82	2013	2013	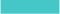  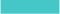 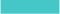 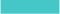 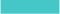 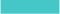 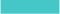 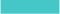 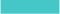 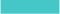 (2012-2022)
	regulatory T cell	1.59	2013	2013	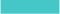  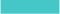 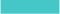 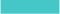 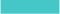 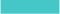 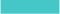 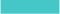 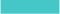 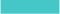 (2012-2022)
	mucosa associated microbiota	1.8	2014	2014	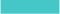 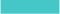  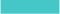 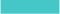 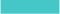 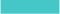 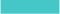 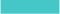 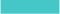 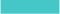 (2012-2022)
	fecal microbiota	1.64	2014	2016	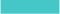 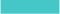    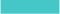 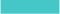 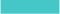 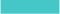 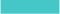 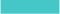 (2012-2022)
	NF kappa B	1.61	2014	2014	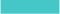 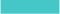  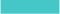 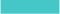 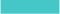 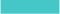 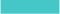 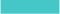 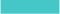 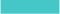 (2012-2022)
	Escherichia coli	1.61	2015	2016	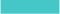 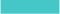 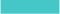   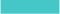 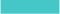 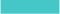 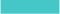 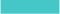 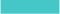 (2012-2022)
	Intestinal microbiota	2.76	2016	2017	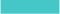 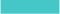 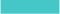 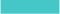   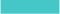 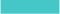 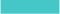 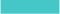 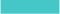 (2012-2022)
	T cell	2.04	2016	2017	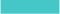 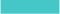 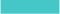 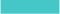   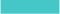 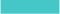 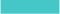 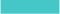 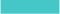 (2012-2022)
	diet induced obesity	1.83	2016	2017	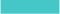 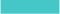 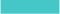 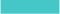   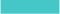 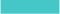 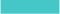 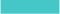 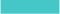 (2012-2022)
	commensal microbiota	1.82	2016	2017	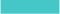 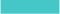 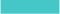 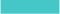   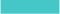 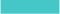 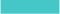 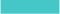 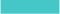 (2012-2022)
	commensal bacteria	1.58	2016	2017	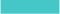 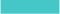 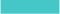 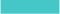   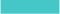 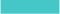 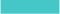 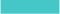 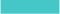 (2012-2022)
	chain fatty acid	2.15	2017	2019	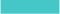 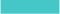 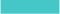 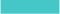 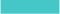    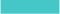 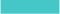 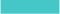 (2012-2022)
	epithelial cell	1.87	2017	2018	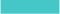 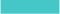 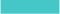 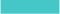 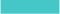   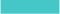 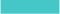 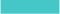 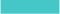 (2012-2022)
	intestinal epithelial cell	1.87	2017	2018	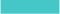 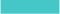 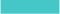 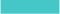 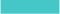   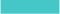 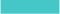 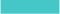 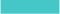 (2012-2022)
	clostridium difficile infection	1.6	2017	2018	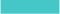 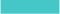 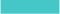 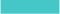 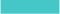   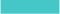 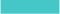 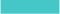 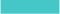 (2012-2022)
	necrosis factor alpha	1.59	2017	2017	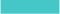 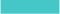 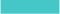 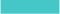 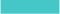  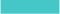 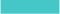 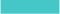 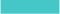 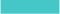 (2012-2022)
	tight junction protein	1.59	2017	2017	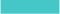 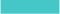 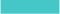 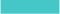 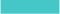  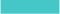 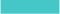 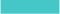 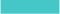 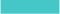 (2012-2022)
	dietary fiber	1.58	2017	2019	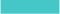 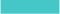 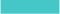 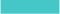 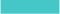    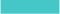 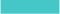 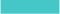 (2012-2022)
	distal ulcerative colitis	1.49	2017	2018	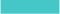 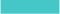 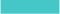 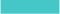 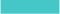   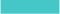 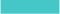 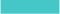 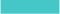 (2012-2022)
	immune system	2.39	2018	2018	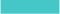 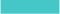 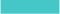 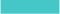 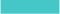 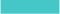  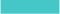 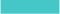 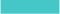 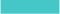 (2012-2022)
	butyrate	1.57	2018	2022	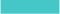 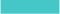 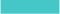 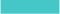 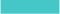 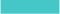      (2012-2022)
	microbiota	2.43	2019	2019	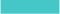 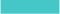 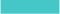 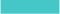 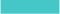 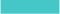 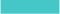  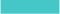 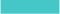 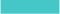 (2012-2022)
	gut microbiota	2.65	2020	2022	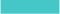 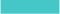 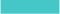 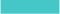 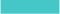 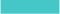 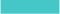 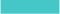    (2012-2022)
	ulcerative colitis	2.16	2020	2020	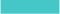 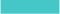 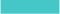 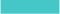 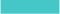 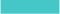 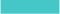 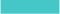  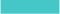 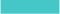 (2012-2022)
	genome wide association	1.85	2020	2020	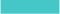 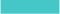 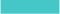 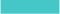 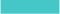 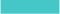 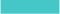 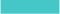  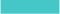 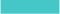 (2012-2022)
	intestinal permeability	1.85	2020	2020	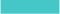 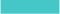 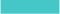 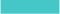 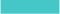 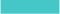 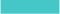 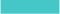  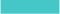 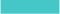 (2012-2022)
	activation	1.65	2020	2022	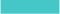 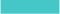 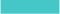 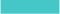 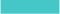 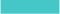 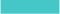 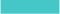    (2012-2022)

A: γ[0,1]=1, Minimum Duration=2; B: γ[0,1]=0.6, Minimum Duration=1.

## 4 Discussion

The climbing global incidence of intestinal inflammatory diseases is a challenging disease and a global burden (Kaplan and Windsor, 2021; Lebwohl and Rubio-Tapia, 2021; Aziz et al., 2022). And the interaction between intestinal flora and immune cells plays a very important role in the pathogenesis of intestinal inflammatory diseases(Hooper et al., 2012). Therefore, the interaction between the microbiota and the immune system in intestinal inflammatory diseases is an essential topic. With the growing body of relevant literature, it is particularly urgent to have an overview of this research area, and bibliometrics can meet this need.

This article was the first based on the bibliometric method combined with the content analysis method to conduct a mathematical statistical analysis of the literature related to the interaction between microbiota and immune in intestinal inflammatory diseases, and to mine research hotspots and development trends based on data information. In general, the number of research publications in this area has been increasing year by year, indicating that this area is relatively popular and promising. The USA and China are the main research-exporting countries. From the perspective of overall global research, the USA is the core country of research in this field, with early research, the largest number of publications, and high centrality of research content, and is in a leading position in the world. Among the top ten countries in terms of the number of publications, China is the only one from a developing country, and it is second only after the USA in terms of the number of publications, which shows that China has made great progress and played an important role in this field over the years, and it also proves that Chinese scholars are approaching and aligning with international research in this field. Although China has made rapid development in this field, it still lacks core achievements with high international influence and leading international research frontiers, and there is a greater need to improve the “quality” of results than to break through the “quantity” of papers. As for the institutions, Harvard University has the largest number of publications, and it has been able to maintain the most extensive collaborative relationships with other institutions and is the most influential institution, in addition to the overall closer collaboration and good cooperative relationships among institutions. Among the highly productive authors, it is noteworthy that Xavier RJ from the Massachusetts Institute of Technology has published not only a high number of papers, but also a high number of citations, and is the author with the highest number of publications in the citation classics. The collaborative network represents the most active and representative research team worldwide and can provide a scientific reference for other scholars conducting relevant research. Xavier RJ is the most widely collaborated author with a high level of influence. But most authors tend to conduct collaborative research within a small group. Therefore, strengthening collaborative research among authors, especially those from different countries or institutions, can greatly facilitate the exchange of scholarly ideas and innovation in the research field. To some extent, the IF of a journal is one of the most powerful indicators of citations and often represents the impact of research in that area. Research in this area is mainly published in Frontiers in Immunology with an IF of 8.787 and Q1, which also reflects the quality of output in this area.

Visualized analysis of keywords in the literature related to the interaction between microbiota and immune in intestinal inflammatory diseases through CiteSpace revealed that the hot words were mainly “inflammatory bowel disease”, “Crohn’s disease”, “ulcerative colitis”, “colorectal cancer”, “gut microbiota”, “intestinal microbiota”, “bacteria”, “immune response”, “regulatory T cell”, “dendritic cell”, “clostridium difficile infection”, “Escherichia coli”, “mucosal immunity”, “chain fatty acid”, “activation” etc. With the deepening and development of research, “fusobacterium nucleatum”, “gut-liver axis”, and “short-chain fatty acid” had become a new emerging topic and future direction. In the early stage of the research, genomics, intestinal bacterial infections, and immune system research were mostly focused on. In the later stage, the research hotspot expanded to the mechanism of intestinal inflammatory diseases. The analysis of the citation classics further confirmed the information above.

Cluster analysis reinforces the fact that the main direction of research in this field is the mechanism of intestinal inflammatory diseases, especially the interaction between the intestinal microbiota and the immune system. It is important for health that the intestinal barrier effectively prevents the invasion and translocation of tubular bacteria and does not overreact to commensal microbes (Mehandru and Colombel, 2021). When the intestinal barrier is compromised, it allows for the more frequent acquisition of microbial antigens by the immature immune system. At this point dendritic cells present in the gut begin to present antigens and T cells, monocytes and macrophages are activated and initiate the production of large amounts of pro-inflammatory cytokines and chemokines. This inflammatory cascade leads to the recruitment of neutrophils, the release of reactive oxygen species, and further intestinal inflammation and necrosis. This inflammation leads to more inflammation and can spread systemically, affecting organs as distant from the brain, which is part of the gut-brain axis (Agirman et al., 2021). Thus, bacterial infection or translocation of the intestine leads to a cascade of immune-inflammatory responses, which in turn leads to apoptosis of IECs and further impairs intestinal barrier function. A vicious cycle of immune inflammation promotes the development of intestinal inflammatory diseases (Xue et al., 2020). It is also noteworthy that excessive signaling and inflammation caused by the recognition of microorganisms by IECs can also promote IBD-associated CRC(Frigerio et al., 2021). T cells and regulatory T cells (Tregs) play an important role in the pathogenesis of intestinal inflammatory diseases because Tregs can suppress the function of effector T cells and promote a more tolerogenic immune phenotype (Xu et al., 2018). In intestinal inflammatory diseases, the balance between T cells and Tregs is altered, when disease progression is promoted by inflammatory cytokine production and T cell activation positive feedback loops (Jacobse et al., 2021). The human intestinal microbiome is an extremely complex micro-ecosystem, with a large number and variety of species, and it involves frequent inter-individual variation and temporal variation in the microbial community with disease progression (Caruso et al., 2020). Also, functional changes associated with microbiome changes are increasingly recognized as important and may become the focus of future research. For example, butyrate, one of the short-chain fatty acids, is an important immunomodulatory molecule in the intestine that improves intestinal barrier integrity, thereby regulating intestinal flora dysbiosis and promoting energy metabolism in the gut-liver axis(Deleu et al., 2021). Bidirectional crosstalk along the gut-liver axis controls gastrointestinal health and disease, and crosstalk is reinforced by parallel increases in liver disease and the prevalence of gastrointestinal and immune disorders (Tilg et al., 2022). Understanding the intricate metabolic interactions between the gut and liver in health and disease opens the way for future targeted therapies. In addition, the study of fusobacterium nucleatum in the intestine is one of the future trends. It has been found that fusobacterium nucleatum is not only associated with various inflammatory diseases (e.g., pneumonia, infective endocarditis, septic liver abscess, pancreatic abscess, IBD, etc.), but also affects the development process of various tumors (e.g., oral cancer, colorectal cancer, etc.)(Brennan and Garrett, 2019). The pathogenesis of fusobacterium nucleatum affecting the development of diseases is not yet fully understood, and a more comprehensive study of the relevant pathogenic mechanisms can be expected in the future. We also hope to develop targeted therapeutic agents and biologics with low toxic side effects for fusobacterium nucleatum, and further, develop standardized and optimized treatment protocols to benefit more patients.

This paper shows that IBD is the most studied and common autoimmune disease of intestinal inflammatory diseases, which is associated with complex interactions between multiple factors including immune inflammation, intestinal microecology, and genetics (Ramos and Papadakis, 2019; Caruso et al., 2020; Chen et al., 2021). Both intrinsic and adaptive immunity are involved in the development of IBD. In IBD, the CD is thought to be caused mainly by the Th1 immune response, characterized by IFN-γ, TNF-α, and IL-12-mediated intestinal inflammation, while UC is associated with Th2 response, with IL-5 and IL-13 dominating in inflammation mediation (Ramos and Papadakis, 2019). Intestinal microecology also plays an important role in the development of IBD (Caruso et al., 2020). Compared to the general population, patients with IBD have intestinal microecological dysbiosis, which is mainly characterized by a decrease in thick-walled bacteria and Bacteroides, and an increase in Aspergillus and Actinomyces (Ramos and Papadakis, 2019). The intestinal flora may impair the intestinal barrier function, activate the immune response, and induce intestinal inflammation through mechanisms such as regulation of IECs apoptosis and synthesis of tight junction-related proteins, leading to the development of IBD (Ramos and Papadakis, 2019). Evidence shows that ecological dysregulation of the gut microbiota is closely associated with CRC (Cheng et al., 2020). Colon cancer cells interact with a microenvironment composed of immune cells, stromal cells and intestinal microbes to suppress or evade the immune response and to shape the immunopathogenesis of CRC (Schmitt and Greten, 2021).

This study has some limitations. Only relevant studies published by WoSCC were included in this study, and the article type was restricted to English literature, which may have ignored high-quality literature in other languages. In addition, the co-citation frequency of the literature is time-dependent, and the total co-citation frequency of the literature published in recent years may be relatively low due to the short publication time, resulting in discrepancies between the study results and the actual situation.

## 5 Conclusions

To the best of our knowledge, this study is the first bibliometric analysis of publications in the field of the interaction between microbiota and immune in intestinal inflammatory diseases using visualization software and data information mining to obtain the current status hotspots and trends in this field, which provides a theoretical basis for its scientific research. Research in this field has focused on the mechanism of intestinal inflammatory diseases, and future research hotspots may be fusobacterium nucleatum, gut-liver axis, and short-chain fatty acid. Relevant researchers can use the results of this study to improve their knowledge and understanding of the field and can be encouraged to explore it further.

## Ethical statement

There is no need for approval of ethics committee or institutional review board, because this study is a bibliometrics study which do not involve any clinical trials and patient consent.

## Data availability statement

The original contributions presented in the study are included in the article/**Supplementary Material**. Further inquiries can be directed to the corresponding authors.

## Author contributions

Conception and design: CL, WS, ZT. Administrative support: JZ, WD. Provision of study materials or patients: CL, WS, ZT. Collection and assembly of data: CL, WS, ZT. Data analysis and interpretation: CL, WS, ZT. Manuscript writing: All authors. All authors contributed to the article and approved the submitted version.
