# Supplementary material for: The interaction between microbiota and immune in intestinal inflammatory diseases: Global research status and trends
Source: Front Cell Infect Microbiol. 2023 Feb 7;13:1128249. doi: 10.3389/fcimb.2023.1128249 (PMC9941562; doi:10.3389/fcimb.2023.1128249)
Supplement: Supplementary file 2 [file Table_2.docx]

Supplementary Table 2. Top 15 ranking table of authors, institutions, and countries based on WoSCC

| Category | Rank | Authors | Number | Institutions | Number | Countries | Number |
| --- | --- | --- | --- | --- | --- | --- | --- |
| All related researches | 1 | Gasbarrini A | 25 | HARVARD UNIVERSITY | 142 | USA | 1131 |
|  | 2 | Sokol H | 25 | INSTITUT NATIONAL DE LA SANTE ET DE LA RECHERCHE MEDICALE INSERM | 109 | CHINA | 707 |
|  | 3 | Heimesaat MM | 23 | HARVARD MEDICAL SCHOOL | 106 | ITALY | 295 |
|  | 4 | Li Y | 23 | UDICE FRENCH RESEARCH UNIVERSITIES | 105 | GERMANY | 280 |
|  | 5 | Sartor RB | 22 | UNIVERSITY OF CALIFORNIA SYSTEM | 104 | ENGLAND | 223 |
|  | 6 | Bereswill S | 21 | INRAE | 78 | CANADA | 221 |
|  | 7 | Wang Y | 20 | CIBER CENTRO DE INVESTIGACION BIOMEDICA EN RED | 60 | FRANCE | 196 |
|  | 8 | Chen Y | 19 | FREE UNIVERSITY OF BERLIN | 59 | SPAIN | 165 |
|  | 9 | Cong YZ | 19 | UNIVERSITY OF NORTH CAROLINA | 59 | JAPAN | 147 |
|  | 10 | Xavier RJ | 19 | CENTRE NATIONAL DE LA RECHERCHE SCIENTIFIQUE CNRS | 58 | AUSTRALIA | 118 |
|  | 11 | Elson CO | 18 | MASSACHUSETTS GENERAL HOSPITAL | 58 | BRAZIL | 114 |
|  | 12 | Langella P | 17 | UNIVERSITY OF TEXAS SYSTEM | 58 | NETHERLANDS | 108 |
|  | 13 | Haller D | 16 | UNIVERSITY OF LONDON | 55 | SOUTH KOREA | 90 |
|  | 14 | Li M | 16 | UNIVERSITE PARIS SACLAY | 53 | SWITZERLAND | 86 |
|  | 15 | Liu Y | 16 | UNIVERSITY OF TORONTO | 51 | SWEDEN | 82 |
| Citation classics | 1 | Xavier RJ | 7 | UNIVERSITY OF CALIFORNIA SYSTEM | 17 | USA | 81 |
|  | 2 | Knight R | 5 | HARVARD UNIVERSITY | 15 | CHINA | 20 |
|  | 3 | Sokol H | 5 | HARVARD MEDICAL SCHOOL | 11 | ENGLAND | 16 |
|  | 4 | Huttenhower C | 4 | BROAD INSTITUTE | 10 | FRANCE | 14 |
|  | 5 | Nunez G | 4 | INSTITUT NATIONAL DE LA SANTE ET DE LA RECHERCHE MEDICALE INSERM | 10 | GERMANY | 13 |
|  | 6 | Pamer EG | 4 | MASSACHUSETTS INSTITUTE OF TECHNOLOGY MIT | 10 | CANADA | 12 |
|  | 7 | Bridonneau C | 3 | INRAE | 8 | ITALY | 11 |
|  | 8 | Gasbarrini A | 3 | UDICE FRENCH RESEARCH UNIVERSITIES | 7 | SCOTLAND | 9 |
|  | 9 | Gevers D | 3 | ASSISTANCE PUBLIQUE HOPITAUX PARIS APHP | 6 | SOUTH KOREA | 8 |
|  | 10 | Kamada N | 3 | HARVARD T H CHAN SCHOOL OF PUBLIC HEALTH | 6 | SWEDEN | 8 |
|  | 11 | Langella P | 3 | HOWARD HUGHES MEDICAL INSTITUTE | 6 | BELGIUM | 7 |
|  | 12 | Lynch SV | 3 | MASSACHUSETTS GENERAL HOSPITAL | 6 | NETHERLANDS | 7 |
|  | 13 | Mazmanian SK | 3 | UNIVERSITY OF ABERDEEN | 6 | SPAIN | 7 |
|  | 14 | Miggiano GAD | 3 | UNIVERSITY OF CALIFORNIA LOS ANGELES | 6 | SWITZERLAND | 7 |
|  | 15 | Ng SC | 3 | UNIVERSITY OF CALIFORNIA SAN DIEGO | 6 | DENMARK | 6 |
