# Supplementary material for: The interaction between microbiota and immune in intestinal inflammatory diseases: Global research status and trends
Source: Front Cell Infect Microbiol. 2023 Feb 7;13:1128249. doi: 10.3389/fcimb.2023.1128249 (PMC9941562; doi:10.3389/fcimb.2023.1128249)
Supplement: Supplementary file 3 [file Table_3.docx]

Supplementary Table 3. Top 15 authors of relevant literature based on CiteSpace

| Rank | All related researches | | | | | Rank | Citation classics | | |
| --- | --- | --- | --- | --- | --- | --- | --- | --- | --- |
|  | Authors | Frequency | Centrality | Degree | Σ |  | Authors | Frequency | Degree |
| 1 | WANG Y | 71 | 0.1 | 43 | 1.44 | 1 | XAVIER R | 4 | 7 |
| 2 | LI Y | 63 | 0.02 | 44 | 1 | 2 | KNIGHT R | 3 | 5 |
| 3 | CHEN Y | 43 | 0.02 | 28 | 1.07 | 3 | GASBARRINI A | 3 | 5 |
| 4 | LIU Y | 43 | 0.01 | 18 | 1.04 | 4 | BRIDONNEAU C | 3 | 12 |
| 5 | ZHANG Y | 36 | 0 | 16 | 1.02 | 5 | RAOUL P | 2 | 5 |
| 6 | LI X | 35 | 0.02 | 42 | 1.11 | 6 | RINNINELLA E | 2 | 5 |
| 7 | ZHANG H | 33 | 0.01 | 37 | 1 | 7 | LIU Z | 2 | 4 |
| 8 | LIU Z | 32 | 0.04 | 42 | 1.16 | 8 | BRAUN J | 2 | 5 |
| 9 | WANG X | 31 | 0.01 | 26 | 1.06 | 9 | CHASSAING B | 2 | 5 |
| 10 | WANG H | 30 | 0.01 | 31 | 1.07 | 10 | KOSTIC A | 2 | 6 |
| 11 | ZHANG Z | 29 | 0 | 16 | 1 | 11 | GEVERS D | 2 | 6 |
| 12 | LIU H | 28 | 0.03 | 38 | 1 | 12 | MELE M | 2 | 5 |
| 13 | ZHANG L | 26 | 0.06 | 28 | 1.28 | 13 | CINTONI M | 2 | 5 |
| 14 | LIU J | 26 | 0.01 | 22 | 1 | 14 | BROWN J | 2 | 4 |
| 15 | LI J | 26 | 0.01 | 27 | 1 | 15 | BROWN E | 2 | 4 |
