# Supplementary material for: The interaction between microbiota and immune in intestinal inflammatory diseases: Global research status and trends
Source: Front Cell Infect Microbiol. 2023 Feb 7;13:1128249. doi: 10.3389/fcimb.2023.1128249 (PMC9941562; doi:10.3389/fcimb.2023.1128249)
Supplement: Supplementary file 4 [file Table_4.docx]

Supplementary Table 4. Top 15 institutions of relevant literature based on CiteSpace

| Rank | All related researches | | | | | | | | Rank | Citation classics | | | |
| --- | --- | --- | --- | --- | --- | --- | --- | --- | --- | --- | --- | --- | --- |
|  | Institutions | Frequency | Institutions | Centrality | Institutions | Degree | Institutions | Σ |  | Institutions | Frequency | Institutions | Degree |
| 1 | Harvard Univ | 63 | NYU | 0.16 | Harvard Univ | 51 | Harvard Univ | 13.21 | 1 | Harvard Univ | 4 | Harvard Univ | 34 |
| 2 | Harvard Med Sch | 46 | Harvard Univ | 0.15 | NYU | 36 | Harvard Med Sch | 1.74 | 2 | Univ Calif San Diego | 3 | Free Univ Berlin | 22 |
| 3 | Univ Toronto | 41 | Univ Toronto | 0.11 | Univ Toronto | 36 | Shanghai Jiao Tong Univ | 1.54 | 3 | Broad Inst MIT & Harvard | 3 | Broad Inst MIT & Harvard | 21 |
| 4 | Massachusetts Gen Hosp | 41 | Karolinska Inst | 0.11 | Harvard Med Sch | 36 | Univ Toronto | 1.44 | 4 | Chinese Univ Hong Kong | 3 | Massachusetts Gen Hosp | 21 |
| 5 | Chinese Acad Sci | 38 | Harvard Med Sch | 0.1 | Massachusetts Gen Hosp | 35 | Univ Calif Los Angeles | 1.4 | 5 | Univ Penn | 2 | Emory Univ | 19 |
| 6 | NYU | 37 | Chinese Acad Sci | 0.08 | Hannover Med Sch | 31 | Univ Milan | 1.38 | 6 | Univ Michigan | 2 | Howard Hughes Med Inst | 17 |
| 7 | Univ N Carolina | 36 | Shanghai Jiao Tong Univ | 0.08 | Univ Calif Los Angeles | 31 | Chinese Acad Sci | 1.37 | 7 | NYU | 2 | Mayo Clin | 14 |
| 8 | McMaster Univ | 36 | Baylor Coll Med | 0.07 | INRA | 30 | Univ N Carolina | 1.31 | 8 | Emory Univ | 2 | NYU | 13 |
| 9 | Univ Michigan | 34 | McMaster Univ | 0.06 | Univ Penn | 30 | INRA | 1.3 | 9 | Univ Aberdeen | 2 | INRA | 13 |
| 10 | INRA | 30 | INRA | 0.06 | Karolinska Inst | 27 | Univ Copenhagen | 1.25 | 10 | Cedars Sinai Med Ctr | 2 | Childrens Hosp Philadelphia | 13 |
| 11 | Univ Sao Paulo | 30 | Hannover Med Sch | 0.06 | McMaster Univ | 27 | INSERM | 1.21 | 11 | MIT | 2 | Univ Aberdeen | 12 |
| 12 | Shanghai Jiao Tong Univ | 29 | Univ Calif Los Angeles | 0.06 | Chinese Acad Sci | 25 | Univ Helsinki | 1.19 | 12 | Mem Sloan Kettering Canc Ctr | 2 | MIT | 12 |
| 13 | China Agr Univ | 29 | Univ Granada | 0.06 | Baylor Coll Med | 23 | Univ Chicago | 1.17 | 13 | CALTECH | 2 | Humboldt Univ | 12 |
| 14 | Hannover Med Sch | 29 | Massachusetts Gen Hosp | 0.05 | Brigham & Womens Hosp | 23 | Univ Cattolica Sacro Cuore | 1.16 | 14 | Harvard Med Sch | 2 | Univ Bern | 12 |
| 15 | Univ Cattolica Sacro Cuore | 28 | Univ N Carolina | 0.05 | INSERM | 22 | Univ Alabama Birmingham | 1.11 | 15 | INRA | 2 | Univ Washington | 12 |
