# Supplementary material for: The interaction between microbiota and immune in intestinal inflammatory diseases: Global research status and trends
Source: Front Cell Infect Microbiol. 2023 Feb 7;13:1128249. doi: 10.3389/fcimb.2023.1128249 (PMC9941562; doi:10.3389/fcimb.2023.1128249)
Supplement: Supplementary file 5 [file Table_5.docx]

Supplementary Table 5. Top 15 countries of relevant literature based on CiteSpace

| Rank | All related researches | | | | | | Rank | Citation classics | | | | | |
| --- | --- | --- | --- | --- | --- | --- | --- | --- | --- | --- | --- | --- | --- |
|  | Countries | Frequency | Countries | Centrality | Countries | Degree |  | Countries | Frequency | Countries | Centrality | Countries | Degree |
| 1 | USA | 1119 | USA | 0.44 | USA | 59 | 1 | USA | 81 | USA | 0.54 | USA | 23 |
| 2 | CHINA | 676 | CHINA | 0.27 | CHINA | 52 | 2 | CHINA | 20 | CHINA | 0.19 | ENGLAND | 16 |
| 3 | ITALY | 290 | ITALY | 0.27 | ITALY | 48 | 3 | ENGLAND | 16 | SWEDEN | 0.12 | CHINA | 15 |
| 4 | GERMANY | 275 | ENGLAND | 0.13 | GERMANY | 47 | 4 | FRANCE | 13 | ENGLAND | 0.11 | FRANCE | 15 |
| 5 | ENGLAND | 220 | GERMANY | 0.11 | ENGLAND | 43 | 5 | GERMANY | 13 | SCOTLAND | 0.11 | SWEDEN | 14 |
| 6 | CANADA | 219 | FRANCE | 0.11 | CANADA | 40 | 6 | CANADA | 12 | SOUTH KOREA | 0.1 | GERMANY | 14 |
| 7 | FRANCE | 193 | SPAIN | 0.09 | FRANCE | 39 | 7 | ITALY | 11 | WALES | 0.09 | SCOTLAND | 13 |
| 8 | SPAIN | 161 | IRAN | 0.08 | SPAIN | 39 | 8 | SCOTLAND | 9 | FRANCE | 0.08 | ITALY | 11 |
| 9 | JAPAN | 144 | CANADA | 0.07 | AUSTRALIA | 37 | 9 | SOUTH KOREA | 8 | GERMANY | 0.05 | NETHERLANDS | 11 |
| 10 | AUSTRALIA | 117 | AUSTRALIA | 0.07 | SWEDEN | 31 | 10 | SWEDEN | 8 | ITALY | 0.05 | CANADA | 9 |
| 11 | BRAZIL | 111 | INDIA | 0.05 | INDIA | 29 | 11 | BELGIUM | 7 | NETHERLANDS | 0.04 | SWITZERLAND | 9 |
| 12 | NETHERLANDS | 107 | CZECH REPUBLIC | 0.04 | NETHERLANDS | 28 | 12 | SWITZERLAND | 7 | CANADA | 0.03 | BELGIUM | 8 |
| 13 | SOUTH KOREA | 89 | GREECE | 0.04 | JAPAN | 27 | 13 | SPAIN | 7 | BELGIUM | 0 | AUSTRALIA | 6 |
| 14 | SWITZERLAND | 85 | RUSSIA | 0.04 | SWITZERLAND | 25 | 14 | NETHERLANDS | 7 | SWITZERLAND | 0 | BRAZIL | 6 |
| 15 | SWEDEN | 80 | CHILE | 0.04 | SINGAPORE | 24 | 15 | JAPAN | 6 | SPAIN | 0 | AUSTRIA | 6 |
