# Supplementary material for: The interaction between microbiota and immune in intestinal inflammatory diseases: Global research status and trends
Source: Front Cell Infect Microbiol. 2023 Feb 7;13:1128249. doi: 10.3389/fcimb.2023.1128249 (PMC9941562; doi:10.3389/fcimb.2023.1128249)
Supplement: Supplementary file 6 [file Table_6.docx]

Supplementary Table 6. Top 10 journals influence of relevant literature based on the Online Analysis platform of Literature Metrology

| Category | Rank | Journals | Total number of articles | Total number of citations | Average number of citations |
| --- | --- | --- | --- | --- | --- |
| Ranked by total number of articles | 1 | FRONTIERS IN IMMUNOLOGY | 213 | 556 | 2.61 |
|  | 2 | INTERNATIONAL JOURNAL OF MOLECULAR SCIENCES | 82 | 91 | 1.11 |
|  | 3 | NUTRIENTS | 76 | 186 | 2.45 |
|  | 4 | INFLAMMATORY BOWEL DISEASES | 75 | 452 | 6.03 |
|  | 5 | PLOS ONE | 73 | 445 | 6.10 |
|  | 6 | FRONTIERS IN MICROBIOLOGY | 68 | 209 | 3.07 |
|  | 7 | WORLD JOURNAL OF GASTROENTEROLOGY | 59 | 312 | 5.29 |
|  | 8 | SCIENTIFIC REPORTS | 49 | 116 | 2.37 |
|  | 9 | GASTROENTEROLOGY | 47 | 995 | 21.17 |
|  | 10 | MUCOSAL IMMUNOLOGY | 43 | 349 | 8.12 |
| Ranked by total number of citations | 1 | PROCEEDINGS OF THE NATIONAL ACADEMY OF SCIENCES OF THE UNITED STATES OF AMERICA | 20 | 1186 | 59.30 |
|  | 2 | NATURE | 15 | 1024 | 68.27 |
|  | 3 | NATURE REVIEWS IMMUNOLOGY | 14 | 1003 | 71.64 |
|  | 4 | GASTROENTEROLOGY | 47 | 995 | 21.17 |
|  | 5 | CELL HOST & MICROBE | 15 | 799 | 53.27 |
|  | 6 | FRONTIERS IN IMMUNOLOGY | 213 | 556 | 2.61 |
|  | 7 | SCIENCE | 9 | 556 | 61.78 |
|  | 8 | INFLAMMATORY BOWEL DISEASES | 75 | 452 | 6.03 |
|  | 9 | PLOS ONE | 73 | 445 | 6.10 |
|  | 10 | GUT | 33 | 411 | 12.45 |
| Ranked by average number of citations | 1 | GENOME BIOLOGY | 1 | 184 | 184.00 |
|  | 2 | ANNUAL REVIEW OF IMMUNOLOGY, VOL 28 | 2 | 198 | 99.00 |
|  | 3 | NATURE REVIEWS IMMUNOLOGY | 14 | 1003 | 71.64 |
|  | 4 | NATURE | 15 | 1024 | 68.27 |
|  | 5 | SCIENCE | 9 | 556 | 61.78 |
|  | 6 | PROCEEDINGS OF THE NATIONAL ACADEMY OF SCIENCES OF THE UNITED STATES OF AMERICA | 20 | 1186 | 59.30 |
|  | 7 | CELL HOST & MICROBE | 15 | 799 | 53.27 |
|  | 8 | LANCET | 3 | 143 | 47.67 |
|  | 9 | NATURE REVIEWS MICROBIOLOGY | 5 | 218 | 43.60 |
|  | 10 | CELL | 9 | 363 | 40.33 |
